# The Role of ARF6 in Biliary Atresia

**DOI:** 10.1371/journal.pone.0138381

**Published:** 2015-09-17

**Authors:** Mylarappa Ningappa, Juhoon So, Joseph Glessner, Chethan Ashokkumar, Sarangarajan Ranganathan, Jun Min, Brandon W. Higgs, Qing Sun, Kimberly Haberman, Lori Schmitt, Silvia Vilarinho, Pramod K. Mistry, Gerard Vockley, Anil Dhawan, George K. Gittes, Hakon Hakonarson, Ronald Jaffe, Shankar Subramaniam, Donghun Shin, Rakesh Sindhi

**Affiliations:** 1 Hillman Center for Pediatric Transplantation of the Children’s Hospital of Pittsburgh of University of Pittsburgh Medical Center (UPMC), Pittsburgh, PA, 15224, United States of America; 2 Department of Developmental Biology and McGowan Institute of Regenerative Medicine, University of Pittsburgh, Pittsburgh, PA, 15261, United States of America; 3 Center for Applied Genomics of the Children’s Hospital of Philadelphia, Philadelphia, PA, 19104, United States of America; 4 Department of Pathology, Division of Pediatric Pathology, Children’s Hospital of Pittsburgh of UPMC, Pittsburgh, PA, 15224, United States of America; 5 Histology Core Laboratory, Children’s Hospital of Pittsburgh of UPMC, Pittsburgh, PA, 15224, United States of America; 6 Department of Internal Medicine, Section of Digestive Diseases, Yale University School of Medicine, New Haven, CT, 06510, United States of America; 7 Paediatric Liver, GI, and Nutrition, King’s College Hospital, London, WC2R 2LS, England; 8 Pediatric General and Thoracic Surgery, Children’s Hospital of Pittsburgh of UPMC, Pittsburgh, PA, 15224, United States of America; 9 Department of Bioengineering, University of California San Diego, La Jolla, CA, 92013, United States of America; 10 Department of Pediatrics and Human Genetics, Children’s Hospital of Pittsburgh of UPMC, Pittsburgh, PA, 15224, United States of America; Texas A&M Health Science Center, UNITED STATES

## Abstract

**Background & Aims:**

Altered extrahepatic bile ducts, gut, and cardiovascular anomalies constitute the variable phenotype of biliary atresia (BA).

**Methods:**

To identify potential susceptibility loci, Caucasian children, normal (controls) and with BA (cases) at two US centers were compared at >550000 SNP loci. Systems biology analysis was carried out on the data. In order to validate a key gene identified in the analysis, biliary morphogenesis was evaluated in 2-5-day post-fertilization zebrafish embryos after morpholino-antisense oligonucleotide knockdown of the candidate gene ADP ribosylation factor-6 (*ARF6*, Mo-*arf6*).

**Results:**

Among 39 and 24 cases at centers 1 and 2, respectively, and 1907 controls, which clustered together on principal component analysis, the SNPs rs3126184 and rs10140366 in a 3’ flanking enhancer region for *ARF6* demonstrated higher minor allele frequencies (MAF) in each cohort, and 63 combined cases, compared with controls (0.286 vs. 0.131, P = 5.94x10^-7^, OR 2.66; 0.286 vs. 0.13, P = 5.57x10^-7^, OR 2.66). Significance was enhanced in 77 total cases, which included 14 additional BA genotyped at rs3126184 only (p = 1.58x10^-2^, OR = 2.66). Pathway analysis of the 1000 top-ranked SNPs in CHP cases revealed enrichment of genes for EGF regulators (p<1 x10^-7^), ERK/MAPK and CREB canonical pathways (p<1 x10^-34^), and functional networks for cellular development and proliferation (p<1 x10^-45^), further supporting the role of EGFR-ARF6 signaling in BA. In zebrafish embryos, Mo-*arf6* injection resulted in a sparse intrahepatic biliary network, several biliary epithelial cell defects, and poor bile excretion to the gall bladder compared with uninjected embryos. Biliary defects were reproduced with the EGFR-blocker AG1478 alone or with Mo-*arf6* at lower doses of each agent and rescued with *arf6* mRNA.

**Conclusions:**

The BA-associated SNPs identify a chromosome 14q21.3 susceptibility locus encompassing the *ARF6* gene. *arf6* knockdown in zebrafish implicates early biliary dysgenesis as a basis for BA, and also suggests a role for EGFR signaling in BA pathogenesis.

## Introduction

Biliary atresia (BA), which is characterized by jaundice and loss of extrahepatic bile ducts (EHBD) at birth, affects 1:18000 Caucasian children and is fatal without surgical intervention [1]. The BA disease phenotype is broad and understanding the molecular and developmental basis of BA can improve diagnosis and selection of treatment. This task poses significant challenges, because BA presents shortly after birth suggesting an origin early during fetal development in the embryologic type of BA, or perinatally in the isolated, potentially infectious/inflammatory type of BA. Either type precludes the elucidation of conclusive evidence. This evidence would ideally document disease progression in sequential fetal tissue. Phenotypic heterogeneity consisting of several associated extrahepatic anomalies further defies either a unified mechanistic hypothesis or a perfect experimental model, but suggests that multiple susceptibility loci may be involved. Extrahepatic manifestations can include gut or cardiovascular anomalies including laterality defects such as asplenia/polyspenia and heterotaxy to varying degrees [2,3]. Disease complexity is compounded further by histological features which can overlap with those of other cholestatic conditions such as neonatal cholestasis, ductal plate malformation, and Caroli’s disease [2,4,5]. A common pathophysiologic finding is failure to excrete bile from the liver on nuclear imaging, mandating surgical drainage with portoenterostomy. This procedure fails in nearly half of all patients leading to bile stasis and cirrhosis. As a result, BA accounts for over a third of all pediatric liver transplants, worldwide [6]. Poorly developed intrahepatic bile ducts can also explain failure of portoenterostomy. Such a lesion cannot be identified with certainty amidst histological sequelae of biliary obstruction in most cases. These features include reactive intrahepatic bile duct proliferation, cholestasis, fibrosis and cirrhosis, which can distort liver architecture beyond recognition [7].

The role of environmental factors is underscored by the association of BA with rotavirus, reovirus, and cytomegalovirus infections, and the fact that these viruses induce comparable lesions experimentally. However, these models do not provide insight into the developmental basis of BA suggested by the presence of ‘minor’ extrahepatic anomalies even in the isolated variety of BA [8,9,10,11].

Toward generating novel unbiased hypotheses with evidence from diseased human subjects, two sets of genome-wide association studies (GWAS) have provided new directions. In two North American children with BA, Leyva-Vega *et al*. [12] observed a common 1.76 heterozygous deletion in chromosome 2q37.3. The deletion was inherited from the father who did not have any liver disease in one of the two affected children, confirming that other loci and/or environmental factors were also necessary in BA pathogenesis. These findings were confirmed by Cui *et al*. in 6 of 61 children with BA from the same investigator group, in whom the deletion was confined to the *GPC1* gene within the same locus [13]. A novel developmental model was used to generate supportive evidence, which consisted of impaired biliary network development after *gpc1* knockdown with morpholino antisense oligonuleotide (MO) in zebrafish embryos. In 339 Chinese children with BA, Cheng *et al*. [14] confirmed the association between BA and an intergenic chromosome 10q24 SNP locus, rs17095355, which had been reported previously by Garcia-Barcelo *et al*. [15] from the same group. This SNP locus indicated the involvement of the neighboring *ADD3* gene in BA, because of differential immunostaining of the ADD3 protein when diseased and normal liver tissue was compared. Recently, Tsai *et al*. [16] also found an association between BA and one of 333 SNPs in the chromosome 10q24 locus in 171 North American BA patients. However, the best associated SNP, rs7099604, was located in the first intron of *ADD3* in this locus. Interestingly, knockdown of *add3* in zebrafish also impaired biliary network formation providing supportive evidence for yet another candidate gene identified with GWAS.

Together, these observations reinforce the idea that multiple susceptibility loci along with environmental factors may be involved in BA, and suggest that replicating a GWAS finding may be challenging. Adding to this difficulty is the fact that the SNPs best associated with BA were located in different regions in the chromosome 10q24 susceptibility locus in Chinese and North American patients. Finally, unrecognized minor extrahepatic anomalies, which are associated even with the ‘isolated’ “non-syndromic” variety of BA, can introduce trait heterogeneity in any test cohort. Unrecognized disease trait heterogeneity is a common cause of failure to replicate an association [17]. These limitations in determining BA pathogenesis with GWAS studies are well served by the availability of a well-characterized zebrafish model of bile duct development. This model can provide supportive evidence for additional novel susceptibility genes also identified by GWAS.

To identify additional susceptibility loci we performed GWAS in Caucasian BA cases and controls who presented to the Children’s Hospital of Pittsburgh (CHP) and the Center for Applied Genomics (CAG) at the Children’s Hospital of Philadelphia. This analysis identified *ARF6* as a leading candidate gene involved in bile duct development, based on SNP associations. To identify other *ARF6*-related pathways members, the top-ranked SNPs identified by GWAS were further evaluated with a systems biology analysis. Similar to previous GWAS studies, we used MO-mediated translation blockade to obtain supportive evidence for the role of *arf6* in the early bile duct development in zebrafish.

## Materials and Methods

Children with BA were enrolled at CHP after obtaining written consent from parents for the University of Pittsburgh IRB approved protocol number 0405628. At CAG, all subjects were enrolled after obtaining written consent from parents per Children’s Hospital of Philadelphia IRB approval number 06–004886. Genotyping of DNA obtained from blood was performed with the Infinium HumanHap550K BeadChip (Illumina, San Diego, CA). SNP genotype frequencies were compared between cases and controls with a chi-square test statistic applied in Plink [18] for SNPs with at least 90% call rate and 1% minor allele frequency (MAF). Fourteen additional patients were genotyped with TaqMan® SNP Genotyping Assay (Life Technologies ID; C_11918520_10) for the candidate SNP; rs3126184. Taqman genotyping assay was not available for rs10140366. All zebrafish experiments were performed with approval from the Institutional Animal Care and Use Committee (IACUC) of the University of Pittsburgh, approval number 13102738.

### ARF6 immunostaining of liver explants from children with BA

Formalin fixed, paraffin embedded (FFPE) sections were stained on the Ventana Benchmark Ultra IHC staining platform using ARF6 polyclonal antibody (Abcam, Cambridge, MA). Antigen retrieval was performed using Ventana’s proprietary ultraCC1 cell conditioning reagent and visualized with the uvDAB detection system. Sections were counterstained with hematoxylin.

### SNP and Pathway Analysis

Among top-ranked SNP loci, we examined enrichment for other genes related to *ARF6* and its associated EGFR pathway genes [19,20,21]. Network and enrichment analyses using Cytoscape, a popular network visualization and analysis tool, and Ingenuity Pathway Analysis (IPA) were performed [22,23]. First, a large human network was created in Cytoscape by integrating protein-protein interaction data from BIOGRID and STRING databases, transcription factor interaction from TRANSFAC, and pathway information from KEGG pathways [24,25,26,27]. From the STRING database that uses a scoring scheme between 0 and 1 based on predicted and experimentally validated protein-protein interactions, a 0.9 cutoff was used to extract highly probable protein-protein interaction. Then, a smaller network was created using only the first neighbors of genes that could be mapped from the top 1000 significant SNPs from the GWAS CHP cohort. For SNP-to-gene mapping, +/- 20kb window was applied on the mapping file provided by the manufacturer for the Infinium HumanHap550K BeadChip (Illumina, San Diego, CA). A total of 2506 genes in the new local network was submitted to Ingenuity Pathway Analysis (IPA) for upstream regulator analysis and enrichment of canonical pathways and biological functional categories.

### Zebrafish strains

Embryos and adult fish were raised and maintained under standard laboratory conditions. We used the following transgenic lines: *Tg(EPV*.*Tp1-Mmu*.*Hbb*:*EGFP)* ^*um14*^ [28], *Tg(EPV*.*Tp1-Mmu*.*Hbb*:*hist2h2l-mCherry)* ^*s93*^ [29], *Tg(fabp10a*:*dsRed;ela3l*:*EGFP)* ^*gz*^ [30], [referred to here as *Tg(Tp1*:*GFP)*, *Tg(Tp1*:*H2B-mCherry)*, and *Tg(fabp10a*:*dsRed)*, respectively], *Tg(ins*:*dsRed)* ^*m1018*^ [31], and *Tg(sox17*:*GFP)* ^*s870*^ [32].

### Morpholino, mRNA, and DNA injections

Embryos were injected at the one—cell stage with 0.5 or 2 ng of *arf6*-ATG MO (5’-GATCTTGGAAAGCATCTTCCCCATG-3’) [33], 3 ng of *arf6a*-UTR MO (5’-GTGCAAGACTTAGTCGCTTTTCCGA-3’) or 3 ng of *arf6b*-UTR MO (5’-GTTCATAAATGGTCAATTCCCTCCA-3’). 120 pg of *arf6a* mRNA or 25 pg of plasmids containing *CMV*:*GFP* constructs was injected into the cell at the one-cell stage.

### Chemical treatment

A 3.17 mM stock of the EGFR inhibitor AG1478 (Cayman Chemical) was prepared in 100% dimethyl sulfoxide (DMSO) and diluted to 1 or 4 μM with egg water. As a control, 0.1% DMSO solution in egg water was used.

### Whole-mount *in situ* hybridization and immunostaining

Whole-mount *in situ* hybridization was performed as previously described [34]. Whole-mount immunostaining was performed as previously described [35], using the following antibodies: anti-GFP (1:1000; Aves Labs, Inc., Tigard, OR), anti-Prox1 (1:1000; Millipore, Billerica, MA), mouse monoclonal 2F11 (1:100; Abcam, Cambridge, MA), anti-Abcb11 (1:200; Kamiya Biomedical, Seattle, WA), anti-dsRed (1:200; Clontech, Mountain View, CA), anti-Arf6 (1:400; Abcam, Cambridge, MA), anti-β-catenin (1:200; Sigma-Aldrich, St. Louis, MO) and Alexa Fluor 488-, 568-, and 647-conjugated secondary antibodies (1:500; Life Technologies, Grand Island, NY). Hoechst 33342 (2.5 ng/ml; Sigma-Aldrich, St. Louis, MO) was used for DNA staining.

### Time-lapse imaging

Time-lapse imaging was performed as previously described [36]. Briefly, *Tg(Tp1*:*GFP);Tg(Tp1*:*H2B-mCherry)* larvae at 74 hpf were partially mounted on 0.5% low-melting agarose in a 35 mm petri dish containing egg water supplemented with 0.2 mM 1-phenyl-2-thiourea (Sigma-Aldrich) and 2 μg/ml tricaine (Sigma-Aldrich). Confocal Z-stack images were captured every 15 minutes for 10 hours using a 20x water dipping lens.

### PED-6 and proliferation assay

PED-6 assay was performed by treating larvae with 0.3 μg/ml PED-6 (Life Technologies) for 3 hours as described previously [37]. Proliferation assay was performed by treating larvae with egg water containing 10 mM EdU (Life Technologies) and 1% DMSO. After one-hour EdU treatment, the larvae were harvested and processed for EdU labeling using the Click-it EdU Imaging kit (Life Technology).

### Generation of GFP expression constructs for morpholino validation

The GFP coding region was amplified by PCR with a forward primer that contains a morpholino target sequence in front of GFP start codon and a reverse primer that contains its stop codon. The PCR products were cloned into the pGEM-T vector (Promega, Madison, WI) and their sequences were analyzed. Clones without any mutations were chosen and used for subcloning into the pCS2^+^ expression vector that contains the CMV promoter and the SV40 polyA site. Plasmids containing the final CMV: GFP constructs were used for injection.

### qRT-PCR methods

Total RNA was extracted from pool of 100 livers of uninjected control and *arf6*-ATG MO-injected larvae at 4.5 dpf by using RNeasy Mini Kit (Qiagen, Valencia, CA). Whole transcriptome amplification system (WTA2) (Sigma Aldrich, St. Louis, MO) was used to synthesize μg quantities of amplified cDNA starting with ~ 50 ng of total RNA. Quantitative PCR was measured in SYBR Green/ROX qPCR master mix (Fermentas, Glen Burnie, MD) with primers (IDT, Coralville, IA) shown in S1 Table. qPCR was performed using 7300 Real Time PCR (Applied Biosystems, Foster City, CA). The relative expression level of genes was shown in fold change as normalized to the house keeping gene, *eef1a1l1* by using the Ct method.

### Image acquisition, processing, and statistical analysis

Zeiss LSM700 confocal and Leica M205 FA epifluorescence microscopes were used to obtain zebrafish image data. Confocal stacks were analyzed using the Zen 2009 software. The length of BEC filopodia and interconnecting bile preductules was measured using the ImageJ software and was shown as means ± SEM (standard error of the mean). The number of BECs was also counted using the ImageJ software as previously described [38]. Unpaired two-tailed Student’s t-test was used for statistical analysis; p<0.05 was considered statistically significant.

## Results

### GWAS identifies *ARF6* as a BA susceptibility locus

**Fig 1:**
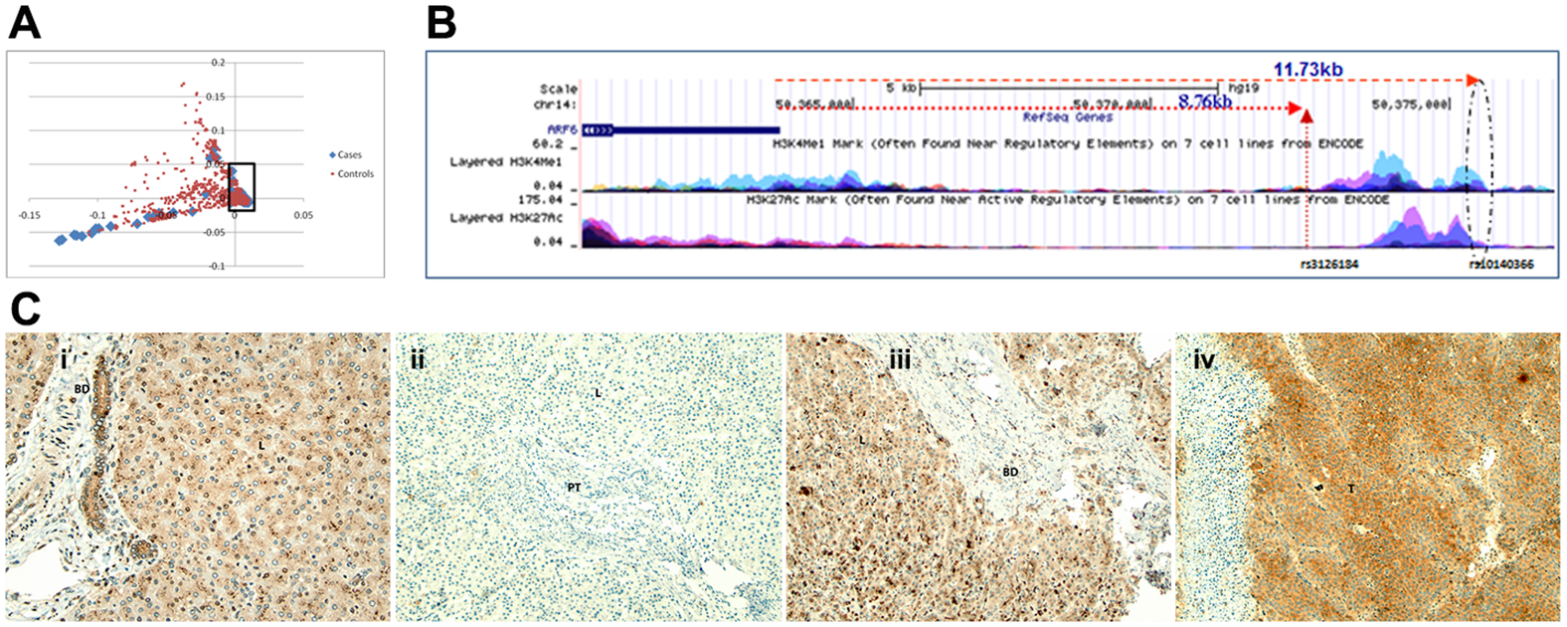
Eigenstrat Principal Components Analysis. (A) Boxed region shows well stratified cases and controls carried forward in analysis. (B) The BA susceptibility locus defined by rs3126184 and rs10140366 lies in an enhancer region in the 3’ flanking region of the *ARF6* gene (UCSC genome browser evaluation show enriched histone marks H3K4me1 overlayed with H3K27Ac is an indicative of enhancer region). (C) i-iv show *ARF6* immunostaining in liver explants from normal children with intact bile ducts (BD) (i), children with BA with bile duct paucity in portal tracts (PT) (ii) or cirrhosis (iii), and a child with hepatocellular carcinoma (iv). T = tumor cells, L = lobule.

All DNA samples had excellent data quality with call rates >98%. Of 50 CHP and 30 CAG BA cases and 2818 controls, 39 CHP and 24 CAG cases, and 1907 controls clustered together on principal component analysis of >550000 SNPs, and demonstrated a very low genomic inflation factor of 1.00627 (Fig 1A) [39]. In comparison of CHP cases with controls, the 1,000 most significant SNPs were ranked further by their proximity to other significant SNPs in the top 1000 in 10 kb windows, to boost confidence in the association (S2 Table). rs3126184 and rs10140366, which ranked 7th and 8th overall in significance emerged as the top-ranked pair demonstrating significantly higher MAFs in CHP cases, in CAG cases, and 63 combined cases from both centers, compared with controls (Table 1). Fourteen additional BA cases genotyped at rs3126184 demonstrated MAF 0.286, and enhanced the significance of the association further, when added to 63 cases above (p = 4.19 x10^-8^, OR = 2.66). rs3126184 and rs10140366 map to 14q22.1 at positions 49442285–49445256 in linkage disequilibrium with each other and the 8.763 kb upstream gene, *ARF6* (Fig 1B). ARF6 regulates embryonic liver development in mice [40]. *ARF6* expression is negatively correlated with the minor alleles of rs1040366 (r = -0.1346, p = 0.023) and rs3126184 (r = -0.1128, p = 0.061) in the combined HapMap populations from SNPexp v1.2 [41]. Together, these findings suggest that ARF6 protein expression may be impaired in BA liver tissue.

**Table 1:** Association testing results for rs3126184 and rs10140366 for 77 BA cases and 1907 controls.

		rs3126184			rs10140366	
	MAF	*p-value	OR	MAF	*p-value	OR
Controls (n = 1907)	0.1308			0.1306		
CHP BA (n = 39)	0.2821	1.03 x10^-4^	2.61	0.2821	9.92 x10^-5^	2.62
CAG BA (n = 24)	0.2917	1.10 x10^-3^	2.74	0.2917	1.10 x10^-3^	2.74
CHP&CAG BA (n = 63)	0.2857	5.94 x10^-7^	2.66	0.2857	5.57 x10^-7^	2.66
CHP BA (n = 14)	0.2857	1.58 x10^-2^	2.66			
ALL BA (n = 77)	0.2857	4.19 x10^-8^	2.66			

*p-value of MAF comparison of the cases to that of the controls.

### GWAS pathway analyses further support the importance of ARF6 and EGFR signaling

Among the 14 SNPs that fall into the coding region of genes, two mapped to the genes, CREB3L4 and CRTC2. These genes are related to cAMP response element-binding protein (CREB) pathway, which can be activated by EGFR signaling through the ERK/MAPK pathway [42,43,44,45]. We also performed network and enrichment analyses using Cytoscape and Ingenuity IPA SNP-to-gene mapping in 20kb+/- windows revealed 299 unique genes associated with 419 of the 1000 to-ranked SNPs (S2 Table). Ingenuity’s upstream regulator analysis on the 2506 genes (S3 Table) from the first-neighbor network created in Cytoscape revealed that out of 632 potential upstream regulators, the 35^th^ ranked regulator, sorted by enrichment p-value, was EGF (p = 1.26 x10^-7^). Other significant EGF-related upstream regulators were TNF (p = 3.14 x10^-25^) and ERK1/2 (p = 3x10^-14^). The significant canonical pathways were ERK/MAPK (p = 5.74 x10^-37^) and cAMP-mediated signaling (p = 1.5 x10^-35^) pathways while enriched functional categories were cellular proliferation (p = 1.85 x10^-103^) and cellular development (p = 1.63 x10^-46^).

### Poor ARF6 staining in human BA liver explants with intrahepatic bile duct paucity

Explants from 29 of 39 CHP cases were immunostained for ARF6 with positive and negative controls. Hepatocytes and bile ducts showed fine granular immunostaining in normal liver tissue (Fig 1Ci). Poor ARF6 staining was seen in two explants with bile duct paucity with minimal fibrosis (Fig 1Cii). In one of these explants, paucity was confined to the left lobe. The right lobe of this and the remaining 27 explants with diffuse ductal hyperplasia, cholestasis, and fibrosis, demonstrated ARF6 staining which was similar to normal liver tissue (Fig 1Ciii). Intense staining was observed in hepatocellular carcinoma (positive control, Fig 1Civ). Therefore, ARF6 protein expression was impaired in the presence of bile duct paucity.

### The zebrafish orthologues of human *ARF6*, *arf6a*, and *arf6b* are expressed in the developing liver and endoderm-derived organs

**Fig 2:**
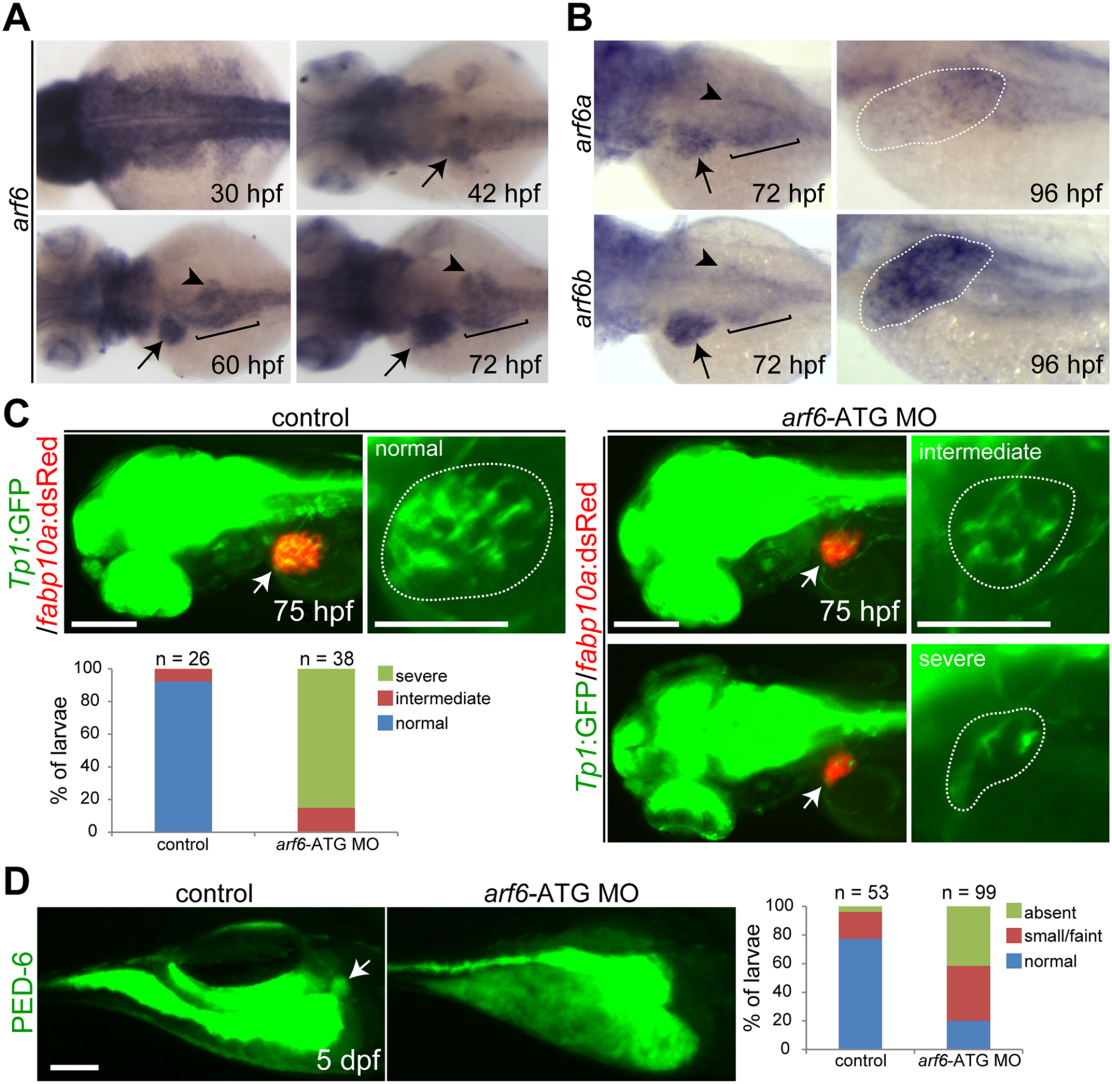
*arf6* knockdown results in developmental biliary defects in zebrafish. (A, B) Whole-mount *in situ* hybridization showing *arf6a* and *arf6b* expression in developing embryos/larvae. Arrows point to the liver; arrowheads the pancreas; brackets mark the intestinal bulb. (C) The *Tg(Tp1*:*GFP)* and *Tg(fabp10a*:*dsRed)* lines reveal the intrahepatic biliary structure and liver size, respectively. Epifluorescence images showing the expression of these transgenes revealed a defect in the intrahepatic biliary structure in *arf6*-ATG MO-injected larvae. Based on the severity of the biliary defect, larvae were divided into three groups: normal, intermediate, and severe. Graph shows the percentage of larvae in each group. Arrows point to the liver; dotted lines outline the liver (A-C). (D) Epifluorescence images showing PED-6 accumulation in the gallbladder (arrows). Based on PED-6 levels in the gallbladder, larvae were divided into three groups: absent, small/faint, and normal. Graph shows the percentage of larvae in each group. n, the number of larvae examined; scale bars, 100 μm.

Two *arf6* genes, *arf6a* and *arf6b*, with identical amino acid sequences, are located on chromosomes 20 and 17, respectively, in zebrafish; their amino acid sequences are 99 percent identical to human ARF6. Using an *in situ* probe for the *arf6a* coding region whose nucleotide sequence is 81% identical to that of *arf6b* (S1 Fig, black box), we found that *arf6a/b* expression appeared ubiquitous at 30 hours post-fertilization (hpf), when the liver-forming region consists of hepatoblasts, but is restricted to the liver at 42 hpf, when hepatoblasts differentiate to biliary epithelial cells (BECs) [46] (Fig 2A, arrows). As embryos further develop, *arf6a/b* expression was clearly observed in the liver and other endoderm-derived organs including the pancreas and the intestinal bulb (Fig 2A). We used *arf6a* and *arf6b in situ* probes targeting non-identical 3’UTR regions of these genes to determine their relative liver expression (S1 Fig, green and red boxes, respectively). Both genes were similarly expressed in the liver and other endoderm-derived organs at 72 hpf (Fig 2B). At 96 hpf, *arf6b* expression persisted whereas *arf6a* expression declined in the liver (Fig 2B).

### *arf6* knockdown results in biliary defects

To investigate the role of both genes in liver development, we designed three translation-blocking antisense morpholino oligonucleotides (MO). *arf6a*-UTR and *arf6b*-UTR MOs targeted non-identical 5’UTR sequences of corresponding genes; *arf6*-ATG MO targeted the *arf6a* ATG start codon region which demonstrated high sequence homology with the start codon for *arf6b* (S1 Fig, underlines). Their efficacy was evaluated by examining green fluorescent protein (GFP) expression from the *CMV*:*GFP* constructs containing each MO-target sequence in front of the *GFP* start codon (S2 Fig). The *arf6*-ATG MO blocked GFP expression from the *CMV*:*GFP* construct containing the corresponding *arf6a* or *arf6b* sequences (S2B Fig) because of high sequence homology in the start codon regions of these two genes, and was therefore used to knock down both genes. The *arf6*-ATG MO was further validated by whole-mount immunostaining with anti-Arf6 antibody. Arf6 expression was greatly reduced in the MO-injected embryos and increased in *arf6a* mRNA-injected embryos (S3 Fig). The *Tg(Tp1*:*GFP)* ^*um14*^ line, which expresses GFP in BEC [46,38], and the *Tg(fabp10a*:*dsRed)* ^*gz15*^ line, which expresses dsRed in hepatocytes [47], were used to evaluate intrahepatic biliary structures and liver size, respectively. Liver size was smaller and importantly intrahepatic biliary ducts were relatively sparse in *arf6*-ATG MO-injected larvae, compared with controls (Fig 2C). These phenotypes were rescued with *arf6a* mRNA in a significant number of larvae (S4A Fig). We next examined whether hepatocyte secretion into bile ducts was defective in *arf6*-ATG MO-injected larvae using the fluorescently labeled fatty acid reporter PED-6, which accumulates in the gallbladder after biliary secretion [37]. PED-6 accumulation in the gallbladder was greatly reduced in the MO-injected larvae compared to controls (Fig 2D). This defect was rescued with *arf6a* mRNA in a significant number of larvae (S4B Fig). Single injection of *arf6a-* or *arf6b*-UTR MO produced biliary defects in a smaller number of larvae than co-injections of these two UTR MOs, or injection with *arf6*-ATG MO (S5 Fig), indicating that both *arf6a* and *arf6b* regulate biliary development.

**Fig 3:**
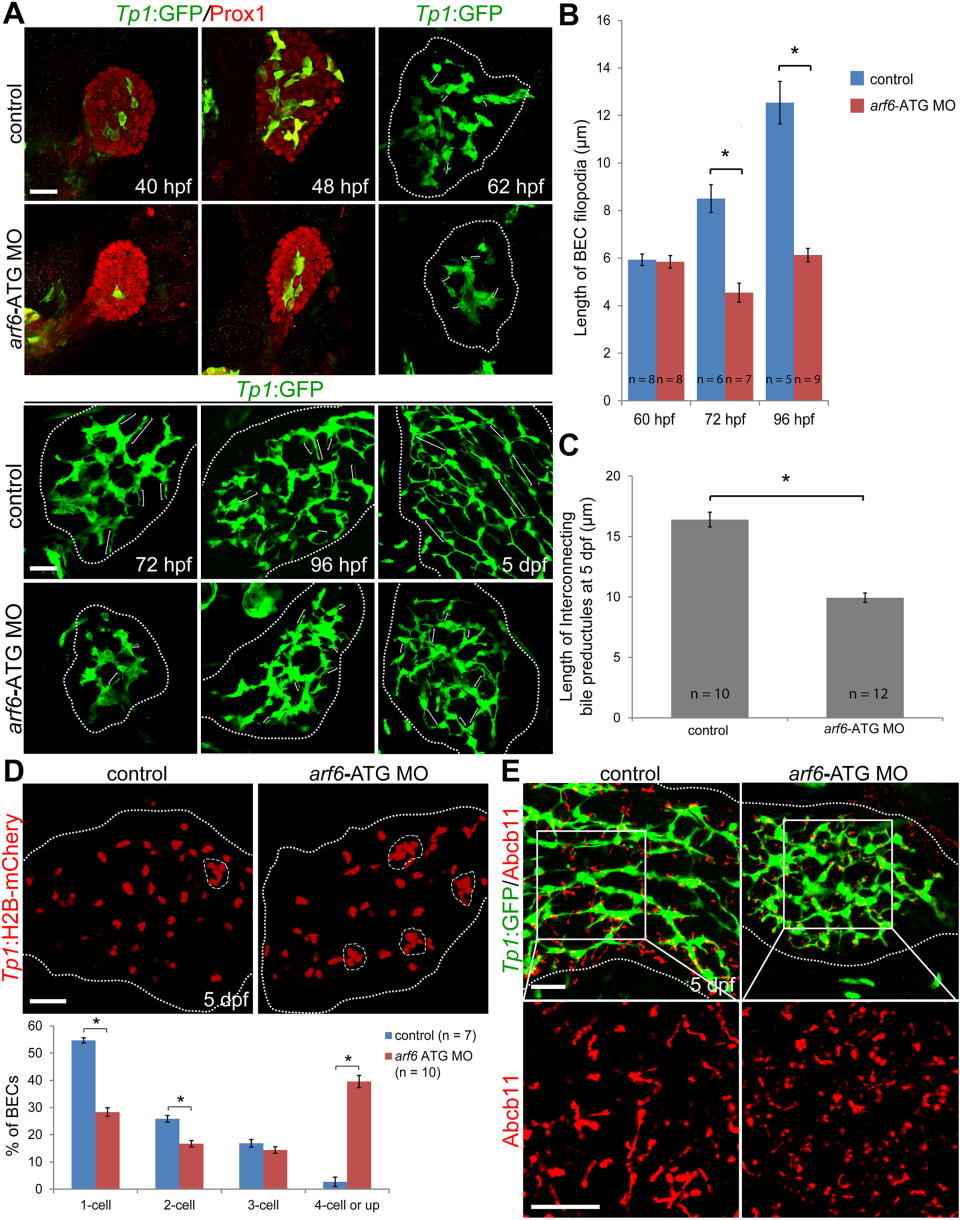
*arf6* regulates intrahepatic biliary morphogenesis. (A-C) Confocal images showing the development of the intrahepatic biliary network. *Tg(Tp1*:*GFP)* embryos/larvae were processed for immunostaining with anti-GFP (green). For 40 and 48 hpf, anti-Prox1 staining (red) was also used. Brackets delineate the length of BEC filopodia at 62, 72, and 96 hpf, and the length of interconnecting bile preductules at 5 dpf (A); graphs show their quantitation (B, C). (D) Confocal images showing the location of BEC nuclei in the entire liver. The *Tg(Tp1*:*H2B-mCherry)* line reveals BEC nuclei. Dashed lines outline clusters with four or more BECs. Graph shows the percentage of BECs present as single cells, doublets, triplets, or in clusters of four or more cells. (E) Confocal images of the liver immunostained for GFP (green) and Abcb11 (red). The *Tg(Tp1*:*GFP)* line and anti-Abcb11 reveal the intrahepatic biliary structure and bile canaliculi, respectively. All dotted lines outline the liver. Asterisks, statistical significance (* p<0.0001); error bars, ± SEM; scale bars, 25 μm.

Detailed characterization of biliary developmental defects was performed at multiple stages from 40 hpf to 5 days post-fertilization (dpf) using whole-mount immunostaining and confocal microscopy. Compared with controls, *arf6*-ATG MO-injected embryos/larvae demonstrated 1) smaller liver size, as assessed by the expression of the hepatoblast marker Prox1 [48] or the hepatocyte marker *fabp10a*:dsRed (Fig 3A), 2) reduced number of BECs, as assessed by *Tp1*:GFP expression, with reduced dispersion and enhanced clustering (Fig 3A), 3) reduced filopodial protrusion of BECs at 72 and 96, but not 62, hpf in fixed larvae (Fig 3B) and from 74 to 84 hpf in live larvae (S6 Fig), 4) reduced length of interconnecting bile preductules at 5 dpf, as represented by the distance between two nuclei of BECs (Fig 3C). In particular, although liver size in the MO-injected embryos at 62 hpf was similar to that in controls at 48 hpf, their BEC distribution was very different from each other, suggesting that the biliary defects observed in the MO-injected embryos were not simply due to developmental delay. We further confirmed the clustering phenotype, using the *Tg(Tp1*:*H2B-mCherry)* ^*s939*^ line that expresses mCherry in BEC nuclei [38]. The quantification of the percentage of single BECs versus cells in cluster of two, three, or four and more cells revealed a significant decrease in the percentage of BECs present as single cells and doublets in the MO-injected larvae compared to controls, concomitant with a significant increase in the percentage of cells in clusters of four and more cells (Fig 3D). This clustering phenotype was not due to increased BEC proliferation. In a proliferation assay, the rate of BEC proliferation was not significantly different between controls and the MO-injected larvae at 75 hpf (S7C Fig), although the total number of BECs in each liver was smaller in the MO-injected larvae than in controls (S7B Fig). Since bile first secretes into bile canaliculi that connect bile preductules in zebrafish and bile ductules in mammals, we also examined the expression patterns of Abcb11, a bile salt export pump present in the bile canaliculi of hepatocytes [49]. The canaliculi were much shorter in the MO-injected larvae than in controls (Fig 3E).

### *arf6* knockdown results in defects in other endoderm-derived organs

Since *arf6a* and *arf6b* are also expressed in other endoderm-derived organs (Fig 2B), we investigated whether other endoderm-derived organs are defective in *arf6*-ATG MO-injected embryos using the *Tg(sox17*:*GFP)* ^*s870*^ line that expresses GFP in all endodermal cells [32]. The pancreas and the intestinal bulb were smaller in the MO-injected embryos than in controls (S8A Fig, arrowheads and brackets, respectively). In addition, the budding of the swim bladder, the zebrafish equivalent of the mammalian lung, was not evident in the MO-injected embryos at 48 hpf, whereas it was evident in controls (S8A Fig, open arrowhead). In contrast to these defects, the gallbladder, EHBD, and the extrapancreatic duct appeared normal in the MO-injected larvae at 75 hpf (S8B Fig), as assessed by the 2F11 antibody, which targets the hepatopancreatic ductal system [35].

### Blocking EGFR signaling results in biliary defects

**Fig 4:**
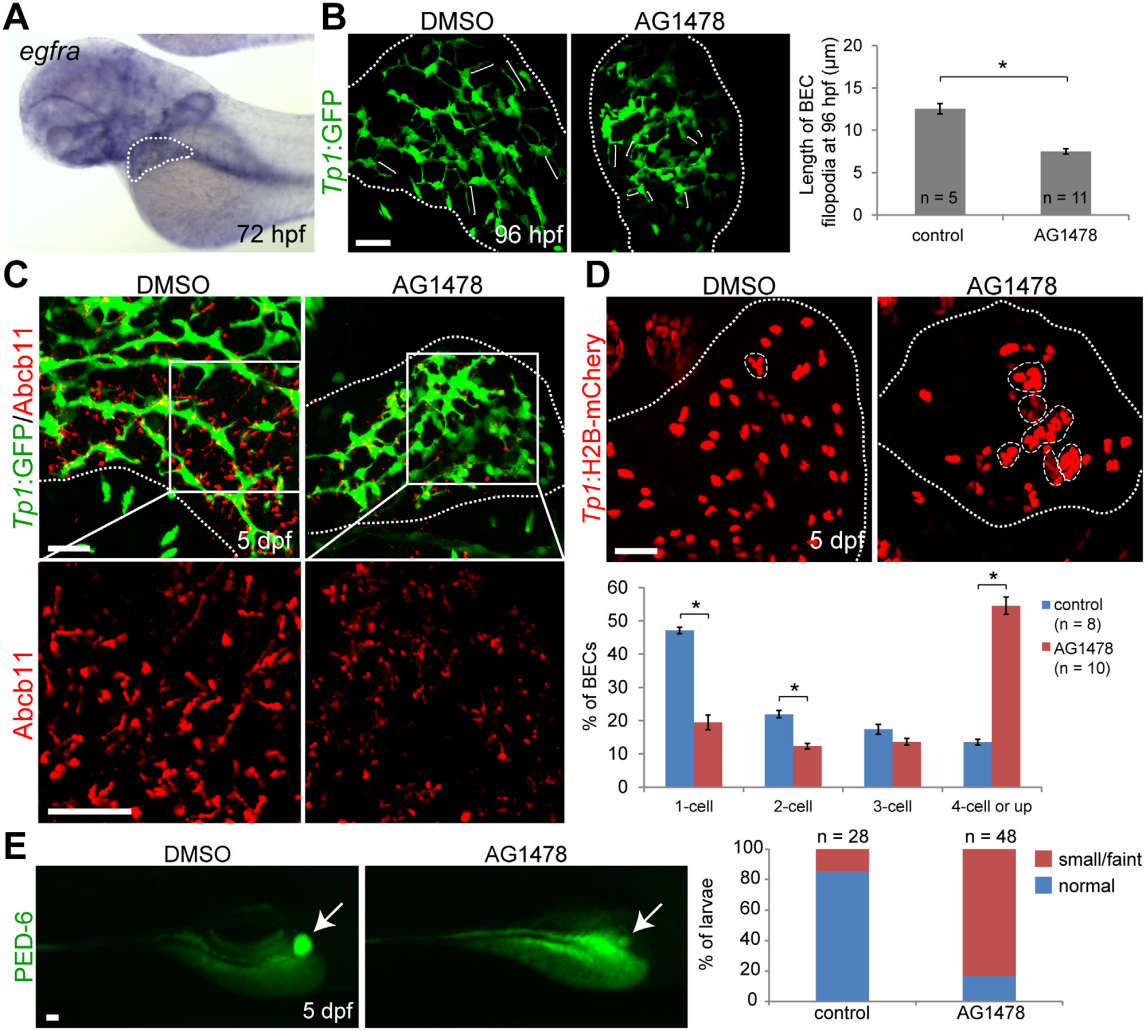
EGFR signaling regulates biliary morphogenesis. (A) Whole-mount in situ hybridization image showing *egfra* expression in the liver at 72 hpf. (B) Confocal images of the liver showing the intrahepatic biliary structure, as revealed by *Tp1*:GFP expression. *Tg(Tp1*:*GFP)* embryos were treated with DMSO or 4 μM AG1478 from 48 to 96 hpf, and processed for whole-mount immunostaining with anti-GFP antibody. The length of BEC filopodia was quantified as shown in a graph. Brackets delineate the length of BEC filopodia. (C) Confocal images of the liver showing the expression of *Tp1*:GFP (green) and Abcb11 (red) for biliary structure and bile canaliculi, respectively. (D) Confocal images of the liver showing the location of BEC nuclei in the entire liver, as assessed by *Tp1*:H2B-mCherry expression. Dashed lines outline clusters with four or more BECs. Graph showing the percentage of BECs present as single cells, doublets, triplets, or in clusters of four or more cells. (E) Epifluorescence images showing PED-6 accumulation in the gallbladder in DMSO- or AG1478-treated larvae at 5 dpf. Graph showing the percentage of larvae exhibiting different levels of PED-6 accumulation in the gallbladder. Arrows point to the gallbladder. All dotted lines outline the liver. n indicates the number of larvae examined; asterisks indicate statistical significance (* p<0.0001). Error bars, ± SEM; scale bars, 25 μm.

Because EGFR signaling activates ARF6 and induces breast cancer invasion, we tested whether impaired EGFR signaling could reproduce the intrahepatic biliary defects observed in *arf6*-ATG MO-injected larvae [50]. The expression of *egfra*, a zebrafish orthologue of human *EGFR*, was clearly observed in the liver at 72 hpf (Fig 4A). Compared with DMSO-treated controls, larvae treated with 4 μM AG1478, a potent EGFR inhibitor [51], from 48 hpf onwards demonstrated 1) reduced BEC filopodial length (Fig 4B), bile canaliculi length (Fig 4C), and PED-6 accumulation in the gallbladder (Fig 4E), 2) enhanced clustering of BECs (Fig 4D) without differences in proliferation at 75 hpf (S7C Fig). Therefore, EGFR-Arf6 signaling pathway may regulate intrahepatic biliary morphogenesis.

**Fig 5:**
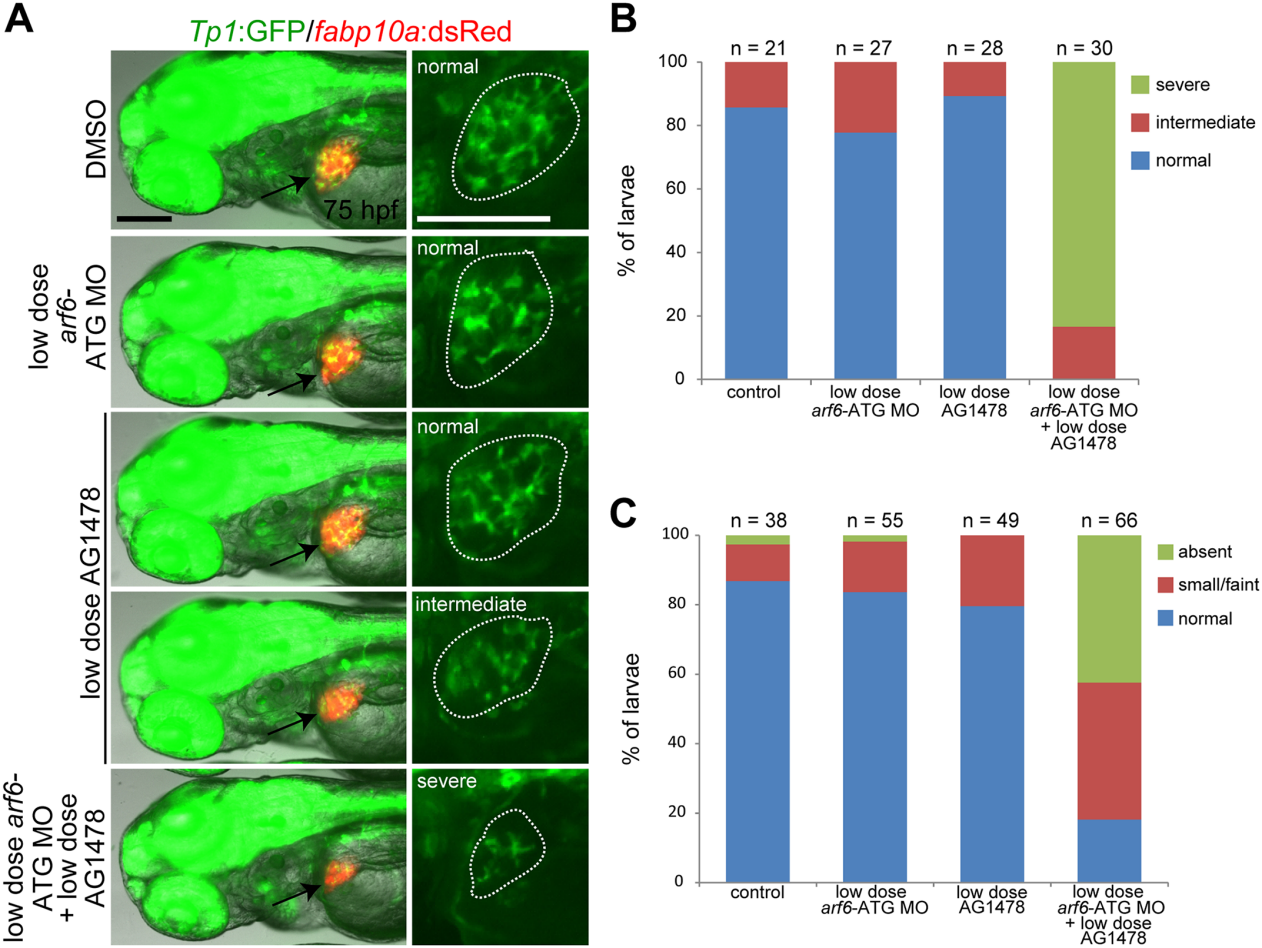
EGFR signaling and Arf6 act in the same pathway in the regulation of intrahepatic biliary morphogenesis. Instead of 4 μM AG1478 and 2 ng of *arf6*-ATG MO, 1 μM AG1478 and 0.5 ng of *arf6*-ATG MO were used. (A) Epifluorescence images showing the expression of *Tp1*:GFP and *fabp10a*:dsRed revealed a severe defect in the intrahepatic biliary structure only when the MO injection was combined with the AG1478 treatment. Based on the severity of the biliary defect, larvae were divided into three groups: normal, intermediate, and severe. Arrows point to the liver and dotted lines outline the liver. Scale bars, 100 μm. (B) Graph showing the percentage of larvae in each group shown in A. (C) Graph showing the percentage of larvae exhibiting different levels of PED-6 accumulation in the gallbladder at 5 dpf. n indicates the number of larvae examined.

We next investigated whether EGFR signaling and Arf6 acted in the same pathway to regulate intrahepatic biliary morphogenesis. At four-fold lower doses (0.5 ng and 1 μM, respectively), *arf6*-ATG MO or AG1478 alone produced minimal or no defects in intrahepatic biliary structure at 75 hpf or in PED-6 accumulation in the gallbladder at 5 dpf. When these two reagents were combined, biliary structure was greatly affected (Fig 5A and 5B) and PED-6 accumulation greatly reduced (Fig 5C), similar to embryos injected with 2 ng of *arf6*-ATG MO (Fig 2C and 2D). These data suggest that EGFR and Arf6 likely regulate intrahepatic biliary morphogenesis via the same pathway.

### EGFR-GEP100-ARF6 pathway genes are upregulated in *arf6* MO-injected larvae

We next examined, by quantitative PCR, the expression levels of EGFR-GEP100-ARF6 pathway genes. ARF6 is necessary for membrane pseudopod formation via ASAP1 [52] and RAC1 and also activates ERK/MAPK signaling via RAC1 [53, 54], We also examined the expression of hedgehog signaling genes which were upregulated in zebrafish embryos after knockdown of *gpc1* in a previous study [13].

**Fig 6:**
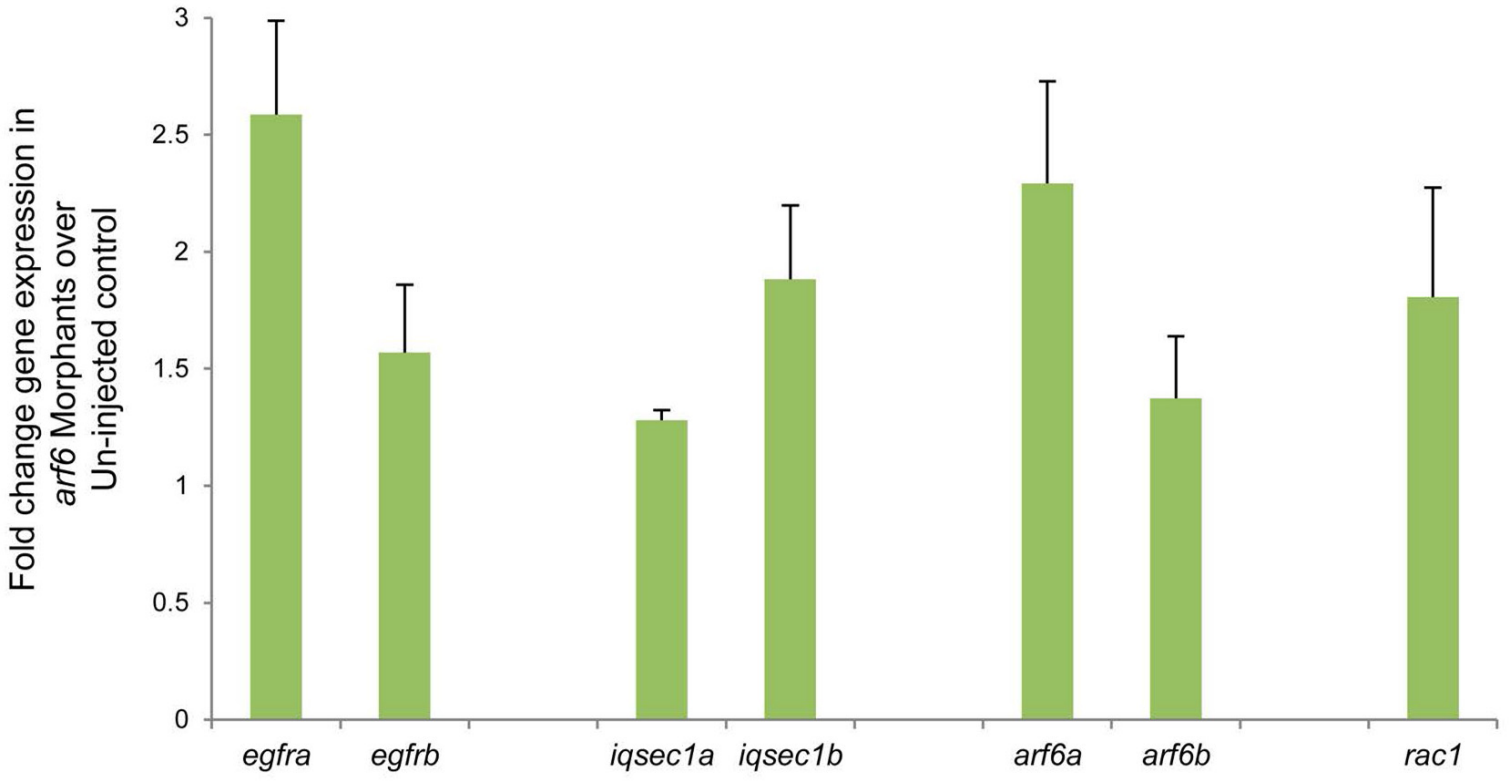
Quantitative RT-PCR analysis of EGFR pathway genes in liver tissue from zebrafish. The relative expression level of genes was shown in fold change in *arf6* morphants over un-injected controls. Error bars shown, ± SEM (average of three independent experiments).

Dissected liver tissue from 4.5-dpf zebrafish larvae, injected with *arf6*-ATG MO in three batches of ~100 larvae each, showed that *egfra*, *gep100*, *arf6*, *asap1 and rac1* were upregulated in all replicates in *arf6* morphants compared with uninjected embryos (Fig 6). Among the hedgehog signaling genes, only *ptch1*, but not *gli1 or gli2a* demonstrated consistent upregulation in all three replicates (S4 Table). The transcription factors, *gli1*, and *gli2a* mediate the effects of the hedgehog receptor *ptch1* on downstream genes in the canonical hehdgehog signaling pathway. These observations suggested that *arf6* knockdown affects EGFR, but not canonical hedgehog signaling in the liver.

## Discussion

Our study identifies the chromosome 14q21.3 locus, defined by the highly associated SNPs rs3126184 and rs10140366, as a susceptibility locus for BA. This 3’ flanking region includes the upstream *ARF6* gene, because minor alleles at both SNP loci are associated with decreased *ARF6* expression in the combined HapMap populations (SNPexp v1.2), and rs10140366 lies in an enhancer region enriched for the histone marks H3K4me1 and H3K27Ac (Fig 1B). Further, *Arf6* ^-/-^ knockout mice show impaired liver development [40]. Although distinct from *GPC1* and *ADD3*, two genes associated with BA in recent GWAS, the *ARF6* gene has similar functional and developmental consequences [13,16]. Knockdown of both *gpc1* and *arf6* in zebrafish impairs biliary network formation. This is expected because GPC1 and ARF6 mediate fibroblast and epidermal growth factor signaling, respectively, which are necessary for the growth and development of organs by epithelial branching morphogenesis [55,56]. ADD3 may also have a developmental role because it is expressed to a greater extent in fetal liver than in adult liver [57]. ARF6 and ADD3 both regulate actin cytoskeletal remodeling, and affect cell mobility and cell-cell contact, which facilitate organogenesis [52,58]. Among the top-ranked 1000 SNPs in our study, rs17127145 closest to the *ADD3* gene ranked 676^th^; none was in proximity to the glypican-1 gene. Therefore, the association between bile duct paucity and poor ARF6 immunostaining in explants with BA led us to further ask whether *arf6* knockdown in zebrafish could affect early biliary morphogenesis and explain the clinical manifestations of BA.

Poor intrahepatic biliary network formation and poor bile excretion manifested as absence of PED-6 accumulation in the gallbladder are dominant effects of *arf6* knockdown. Clinical correlates include failure to excrete radionuclide into the extrahepatic bile duct on hepatobiliary scanning, failure of surgical drainage, and bile duct paucity in some BA patients [59,60]. In *arf6*-ATG MO-injected zebrafish embryos, poor bile excretion also results from reduced biliary canalicular length and enhanced clustering of BEC, presumably due to reduced BEC mobility during morphogenesis. Altered epithelial cell polarity is a known effect of ARF6 deficiency in MDK canine kidney cell line [61]. Clinical correlates include canalicular dilatation with cystic bile lakes characteristic of the “ductal plate malformation” in up to a fourth of BA patients [62]. Because MO is diluted out by cell division, *arf6* MO effects are short-lived. *arf6* mutant lines are needed to investigate long-term consequences. Other pathways may also function at subsequent developmental stages and explain why EHBD loss and atretic gallbladders characteristic of human BA were absent in *arf6* MO-injected zebrafish.

**Fig 7:**
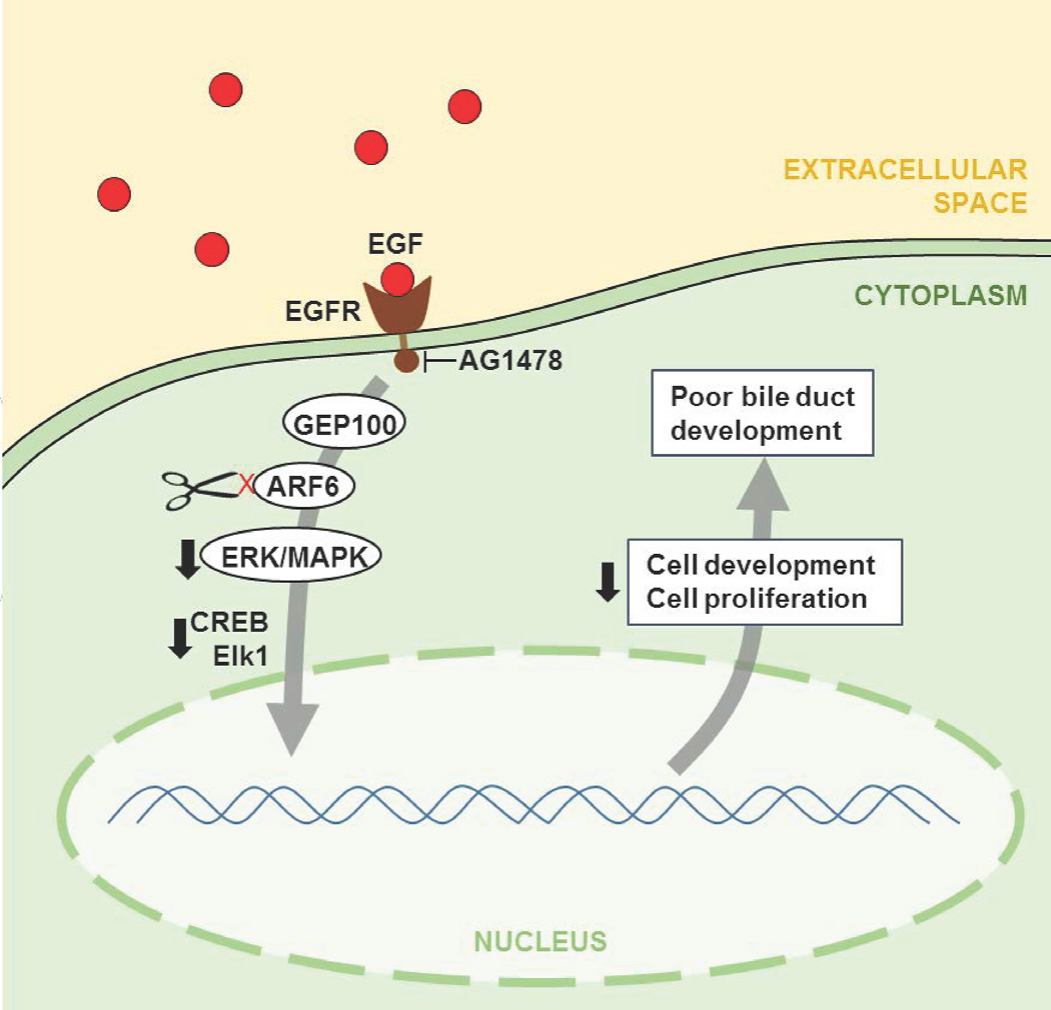
A proposed mechanism for poor bile duct development in BA. Ligation of activated EGFR to GEP100 initiates sequential activation of ARF6, ERK/MAPK and CREB signaling proteins resulting in cellular development and proliferation. Negative regulation of *ARF6* originating from the regulatory regions defined by rs3126184 and rs10140366 in human BA, and *arf6* knockdown or EGFR inhibition with AG1478 in zebrafish embryos lead to poor bile duct development.

ARF6 is a critical member of the EGFR pathway, which facilitates organ development, growth and regeneration by regulating epithelial branching morphogenesis [56]. ARF6 is activated by the binding of EGFR to its activator, GEP100 and has several downstream effects. Activation of the downstream effector ASAP1 is necessary for a normal actin cytoskeleton and cell membrane function [52]. An upregulated EGFR-GEP100-ARF6 pathway facilitates invadopodia formation in invasive breast cancer. ARF6 is also required for RAC1-mediated epithelial cell polarity [61] and membrane pseudopod formation [53]. RAC1 activation also regulates ERK signaling [54]. Sequential activation of ERK/MAPK and CREB signaling pathways is required for normal cellular development and proliferation, and is another downstream effect of EGFR-GEP100-ARF6 signaling [43,44,45,52]. Systems biology analysis of GWAS results supports the role of this molecular pathway in BA by showing enrichment of EGF regulatory genes, the canonical ERK/MAPK and CREB signaling pathways, and the functional categories of cellular development and proliferation (Fig 7).

Therefore, we hypothesized further and found that EGFR signaling blockade with the inhibitor AG1478 reproduced all biliary defects associated with *arf6*-ATG MO-induced knockdown in zebrafish. Further, reduced doses of AG1478 and *arf6*-ATG MO also reproduced these defects when used in combination, but not when used alone. These observations suggest that EGFR-ARF6 signaling regulates early branching morphogenesis of the biliary tree, and that functionally impaired pathway members could also contribute to poor bile drainage likely from abnormal intrahepatic biliary network in children with BA. Supportive evidence consists of poor ARF6 immunostaining in those liver explants, which manifested bile duct paucity without fibrosis, but not in the remainder, in which advanced cirrhosis with marked cholestasis precluded an evaluation of intrahepatic bile ducts prior to the development of chronic obstructive changes (Fig 1C).

The complex molecular effects of *arf6* knockdown in zebrafish also provide potential explanations for the fibrosis that is seen in BA. *arf6* knockdown in zebrafish was associated with intrahepatic upregulation of *egfra*, *gep100*, *arf6*, and genes for downstream effectors of Arf6 such as *asap1 and rac1* (Fig 6). Among the canonical hedgehog signaling pathway genes, which have been implicated previously in biliary dysgenesis only *ptch1*, which induces several hedgehog target genes via effects on the gli family of receptors, and not the *gli1* or *gli2* genes, was consistently upregulated in *arf6* morphants (S4 Table) (13). Activation of Erk by sonic hedgehog can occur independent of canonical hedgehog signaling via PTCH [63] further confirming that ARF6 dysregulation may have several downstream effects on cell membrane function, cell proliferation and cell development via effects on downstream effectors, ASAP1, RAC1 and PTCH1. In experimental models, upregulated EGFR pathway members lead to fibrosis and cirrhosis, a common finding at liver transplantation after failed portoenterostomy for BA [64].

These observations from an established zebrafish model of bile duct development provide additional supportive evidence for the biological relevance of ARF6 in biliary atresia in this first report. Obvious future directions also include replication of these experimental findings. This task requires that the effects of an induced perturbation be observed over several days in invitro mammalian cell lines, because complete disruption of the *ARF6* gene in Arf6 -/- mice leads to almost complete lethality [40]. Because previous studies conducted in patients with BA indicate a high likelihood of multiple susceptibility loci and the environment in disease pathogenesis, future models, which cannot demonstrate such additive effects are likely to maintain a supportive role placing the larger burden of proof back on the human evidence from diseased subjects.

It is generally assumed that the intrahepatic biliary tree is also abnormal in extrahepatic atresia [65,59,60]. However, intrahepatic ducts cannot be examined before the onset of jaundice and clinical evidence of extrahepatic obstruction in human BA. Anecdotally, serial biopsies from four children with confirmed extrahepatic biliary atresia have demonstrated that intrahepatic bile duct paucity without fibrosis preceded obstructive ductal proliferation and cirrhosis at portoenterostomy or liver transplantation [66]. Features indicative of intrahepatic duct involvement are predominantly clinical; disease progression despite portoenterostomy, an attenuated intrahepatic picture on cholangiography; and histopathologic—lymphocytes around and within intrahepatic BECs [67,68]. In the Rhesus rotavirus mouse model of BA, intrahepatic portal areas may contain lymphocytes though the relevance of the model to human BA is unclear [11,69]. Also not evident is whether intrahepatic changes in human BA are primary, or secondary to extrahepatic obstruction. Landing, in 1974, proposed a unitarian hypothesis of infantile obstructive cholangiopathies in which the entire biliary tree was vulnerable but extrahepatic or intrahepatic disease depended on the developmental timing and nature of the insult [70]. The concept lapsed for lack of proof until now. The zebrafish model from two independent studies, including ours, suggests again that the entire biliary tree is genetically susceptible and that in this instance, the outcome of *ARF6* dysregulation involves the intrahepatic tree.

In summary, GWAS studies and analysis of SNP data for functions and pathways postulates the role of ARF6, involved in the EGFR pathway, in defective bile duct formation. We validate this hypothesis in a zebrafish model, which unambiguously shows that *arf6* knockdown impacted the EGFR pathway in the liver and lead to defective bile duct formation.

## Supporting Information

S1 Fig Sequence alignment between *arf6a* and *arf6b* cDNA. Sequences chosen for the design of *arf6*-ATG, *arf6a*-UTR, and *arf6b*-UTR MOs are underlined black, green, and red, respectively. The target regions of *arf6*, *arf6a*, and *arf6b* in situ probes are boxed black, green, and red, respectively. The start (ATG) and stop (TAA) codons of these two genes are red colored.
(TIF)

S2 Fig Morpholino validation. (A-D) *CMV*:*GFP* constructs containing the target sequence of each MO in front of the GFP start codon were injected alone or together with the corresponding MO. GFP expression was barely detected in the co-injected embryos. Graphs show the percentage of embryos exhibiting strong, weak, or no GFP expression. *arf6*-ATG MO also blocked GFP expression from the *CMV*:*GFP* constructs containing the *arf6b* region corresponding to the MO target region in *arf6a* (B), indicating that *arf6*-ATG MO blocks both *arf6a* and *arf6b* translation. n indicates the number of embryos examined. Scale bars: 200 μm.
(TIF)

S3 Fig Arf6 expression is greatly reduced in *arf6*-ATG MO-injected embryos. Wild-type embryos were injected at the one-cell stage with 2 ng of *arf6*-ATG MO or 120 pg of *arf6a* mRNA, harvested at 7 hpf, and processed for whole-mount immunostaining with anti-Arf6 (green) and anti-β-catenin (red) antibodies. DNA was also stained with Hoechst 33342 (gray). β-catenin expression reveals the cell membrane. Confocal images showed that Arf6 expression was greatly increased in *arf6a* mRNA-injected embryos, whereas it was greatly reduced in *arf6*-ATG MO-injected embryos, validating the efficacy of the MO. Dorsal views; scale bar, 25 μm.
(TIF)

S4 Fig *arf6a* mRNA injection partially rescues biliary defects in *arf6*-ATG MO-injected larvae. (A) The *Tg(Tp1*:*GFP)*, *Tg(fabp10a*:*dsRed)*, and *Tg(ins*:*dsRed)* lines were used to reveal the intrahepatic biliary structure, the liver, and the dorsal pancreas, respectively. Epifluorescence images showing the expression of these transgenes revealed that a defect in the intrahepatic biliary structure in *arf6*-ATG MO-injected larvae was partially rescued by *arf6a* mRNA injection. Based on the severity of the biliary defect, larvae were divided into three groups: normal, intermediate, and severe. Graph showing the percentage of larvae in each group. Arrows point to the liver; arrowheads point to the dorsal pancreas. Dotted lines outline the liver. Lateral views, anterior to the left. (B) Epifluorescence images showing PED-6 accumulation in the gallbladder revealed that the PED-6 accumulation defect in *arf6*-ATG MO-injected larvae was also partially rescued by *arf6a* mRNA injection. Based on PED-6 levels in the gallbladder, larvae were divided into three groups: absent, small/faint, and normal. Graph showing the percentage of larvae in each group. Arrows point to the gallbladder. Lateral views, anterior to the right. n indicates the number of larvae examined. Scale bars, 100 μm.
(TIF)

S5 Fig Simultaneous knockdown of *arf6a* and *arf6b* results in more severe biliary defects than their single knockdown. (A) The *Tg(Tp1*:*GFP)*, *Tg(fabp10a*:*dsRed)*, and *Tg(ins*:*dsRed)* lines were used to reveal the intrahepatic biliary structure, the liver, and the dorsal pancreas, respectively. Epifluorescence images showing the expression of these transgenes revealed that a severe biliary defect was observed more often in larvae co-injected with 3 ng of *arf6a*-UTR and 3 ng of *arf6b*-UTR MOs than in singly injected larvae. Based on the severity of the biliary defect, larvae were divided into three groups: normal, intermediate, and severe. Graph showing the percentage of larvae in each group. Arrows point to the liver; arrowheads point to the dorsal pancreas. Dotted lines outline the liver. Lateral views, anterior to the left. (B) Epifluorescence images showing PED-6 accumulation in the gallbladder. Based on PED-6 levels in the gallbladder, larvae were divided into three groups: absent, small/faint, and normal. Graph showing the percentage of larvae in each group. Arrows point to the gallbladder. Lateral views, anterior to the right. n indicates the number of larvae examined. Scale bars, 100 μm.
(TIF)

S6 Fig The movement of BEC filopodia is greatly reduced in *arf6*-ATG MO-injected larvae. (A, B). Time-lapse confocal images showing BEC behaviors in *arf6*-ATG MO-injected and control larvae. The behaviors were assessed by *Tp1*:GFP (green) and *Tp1*:H2B-mCherry (red) expression in BEC cytoplasm and nuclei, respectively. Confocal images every 60 minutes from 74 to 84 hpf were presented. Arrows point to BEC filopodia. Scale bars, 25 μm.
(TIF)

S7 Fig Proliferation is not affected in *arf6*-ATG MO-injected or AG1478-treated larvae at 75 hpf. (A) Confocal images showing EdU^+^ proliferating cells (gray) and H2B-mCherry^+^ BECs (red) in the liver of control, *arf6*-ATG MO-injected, or AG1478-treated *Tg(Tp1*:*H2B-mCherry)* larvae. For EdU labeling, the larvae were treated with EdU for one hour prior to harvest. Dotted lines outline the liver. Scale bar, 50 μm. (B) Graph showing the total number of BECs in each liver. Asterisks indicate statistical significance: * p<0.0001, ** p<0.005. (C) Graph showing the percentage of EdU^+^ BECs among BECs. Error bars, ± SEM. n indicates the number of larvae examined.
(TIF)

S8 Fig The effect of *arf6* knockdown on the development of endoderm-derived organs. (A) Confocal images showing the endoderm and endoderm-derived organs at 48 hpf. The *Tg(sox17*:*GFP)* line was used to reveal the endoderm and endoderm-derived organs. Arrows, arrowheads, and open arrowhead point to the liver, the pancreas and the swim bladder, respectively; brackets mark the intestinal bulb. (B) Confocal images showing the hepatopancreatic ductal system at 75 hpf. *Tg(Tp1*:*GFP);Tg(fabp10a*:*dsRed)* larvae were processed for whole-mount immunostaining with 2F11 (gray), GFP (green), and dsRed (red) antibodies. In contrast to the intrahepatic biliary defect, the hepatopancreatic ductal system, revealed by 2F11 antibody, appeared to be normal in *arf6*-ATG MO-injected larvae. Arrows, arrowheads, open arrowheads, and open arrows point to the gallbladder (gb), the extrahepatic duct (ehd), the common bile duct (cbd), and the extrapancreatic duct (epd), respectively. The hepatopancreatic ductal system is schematically illustrated. Ventral views, anterior up. Scale bars, 50 μm.
(TIF)

S1 Table qPCR primer sequences for gene expression studies in liver tissue from zebrafish. (DOCX)

S2 Table Four hundred nineteen SNPs mapped to unique 299 genes within 20kb+/- windows among the top 1000 significant SNPs in CHP cohort with BA in the genome wide association study. (XLS)

S3 Table Among the 1000 top-ranked significant SNPs, 419 mapped to 299 unique genes as described in the methods. Among these genes, 231 showed interaction with other genes from the large human network consisting mainly of protein-protein interaction. The first neighbor network was created by assimilating all of the first interacting genes with these 231 genes from the large human network. The total of 2506 genes in the new smaller network is listed in the table. More information can be found in the table that includes the average P-values calculated from multiples SNPs and the interacting genes.
(XLSX)

S4 Table Quantitative RT-PCR analysis of hedgehog target genes in liver tissue from zebrafish. (DOCX)
