# Supplementary figures and images for: The Role of ARF6 in Biliary Atresia
Source: PLoS One. 2015 Sep 17;10(9):e0138381. doi: 10.1371/journal.pone.0138381 (PMC4574480; doi:10.1371/journal.pone.0138381)

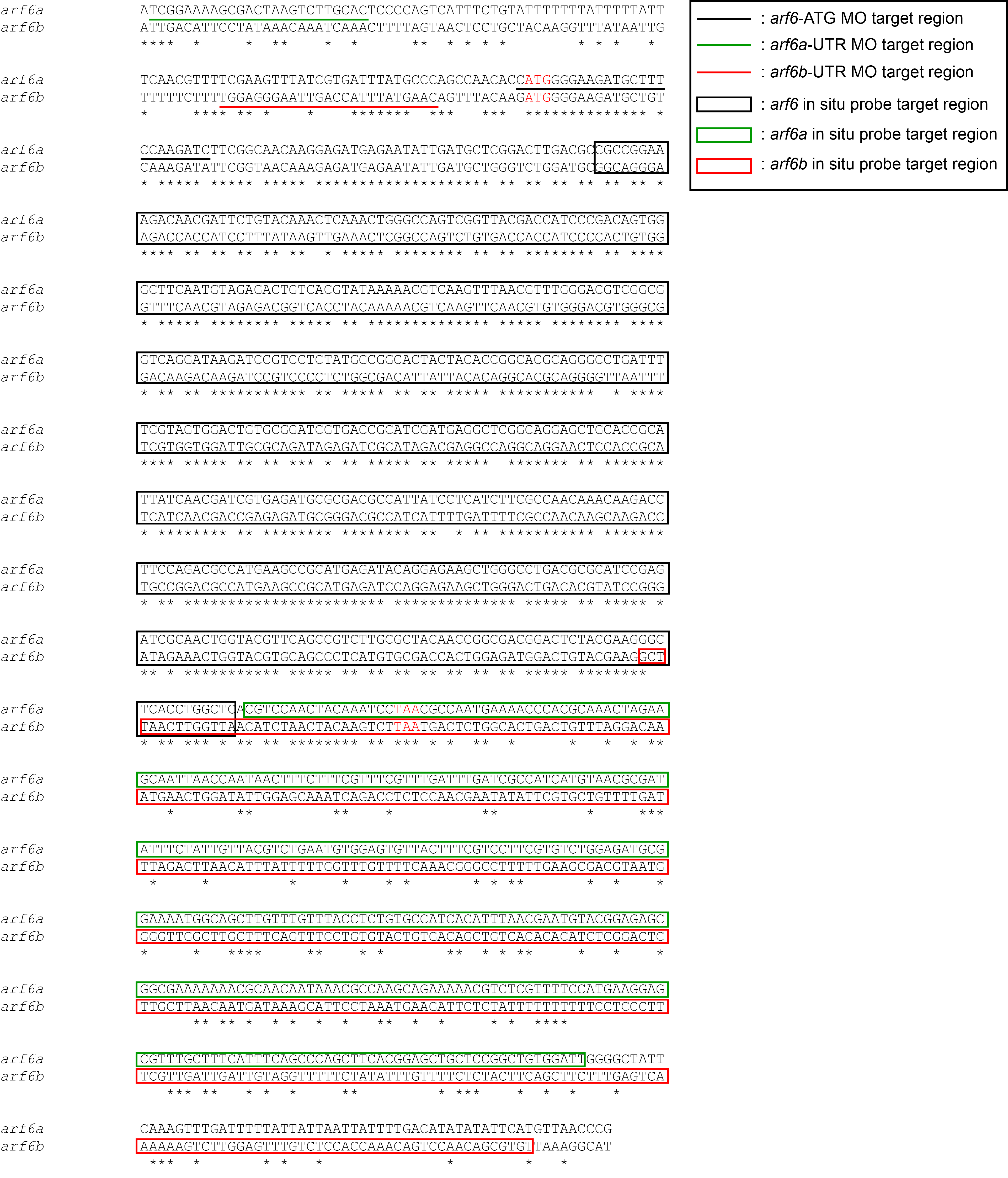

Supplement: S1 Fig — Sequences chosen for the design of arf6-ATG, arf6a-UTR, and arf6b-UTR MOs are underlined black, green, and red, respectively. The target regions of arf6, arf6a, and arf6b in situ probes are boxed black, green, and red, respectively. The start (ATG) and stop (TAA) codons of these two genes are red colored. (TIF) [file pone.0138381.s001.tif]

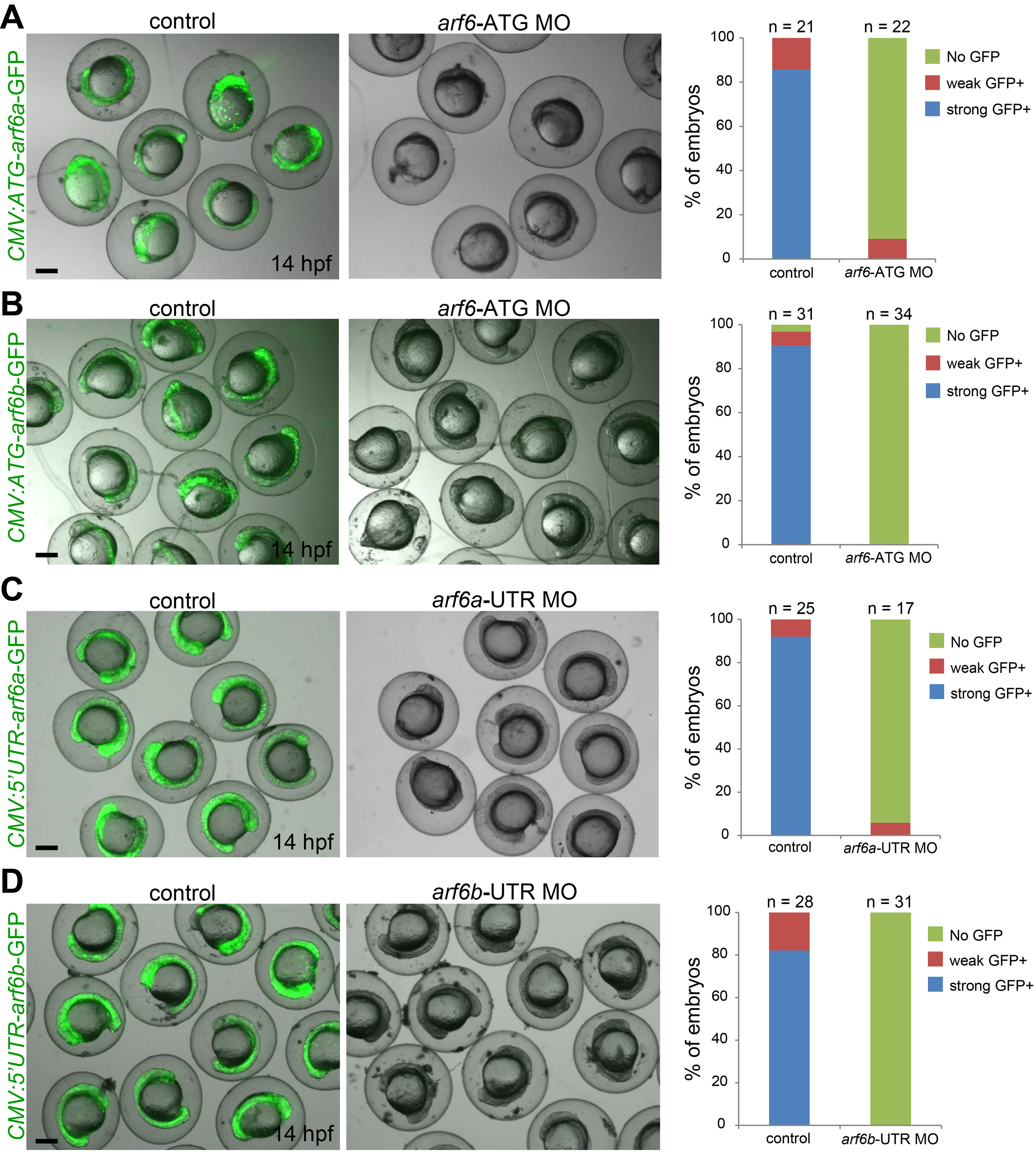

Supplement: S2 Fig — (A-D) CMV:GFP constructs containing the target sequence of each MO in front of the GFP start codon were injected alone or together with the corresponding MO. GFP expression was barely detected in the co-injected embryos. Graphs show the percentage of embryos exhibiting strong, weak, or no GFP expression. arf6-ATG MO also blocked GFP expression from the CMV:GFP constructs containing the arf6b region corresponding to the MO target region in arf6a (B), indicating that arf6-ATG MO blocks both arf6a and arf6b translation. n indicates the number of embryos examined. Scale bars: 200 μm. (TIF) [file pone.0138381.s002.tif]

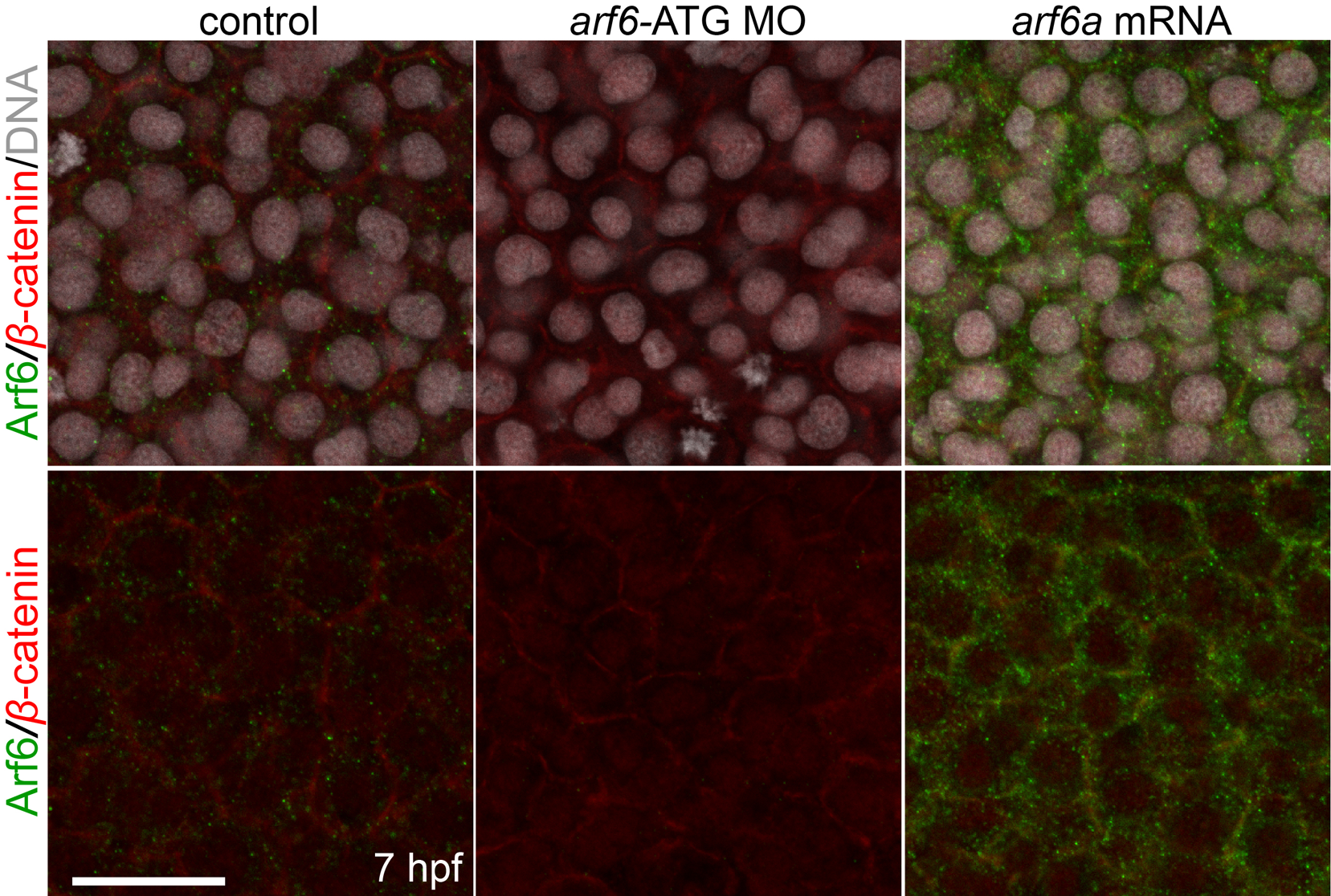

Supplement: S3 Fig — Wild-type embryos were injected at the one-cell stage with 2 ng of arf6-ATG MO or 120 pg of arf6a mRNA, harvested at 7 hpf, and processed for whole-mount immunostaining with anti-Arf6 (green) and anti-β-catenin (red) antibodies. DNA was also stained with Hoechst 33342 (gray). β-catenin expression reveals the cell membrane. Confocal images showed that Arf6 expression was greatly increased in arf6a mRNA-injected embryos, whereas it was greatly reduced in arf6-ATG MO-injected embryos, validating the efficacy of the MO. Dorsal views; scale bar, 25 μm. (TIF) [file pone.0138381.s003.tif]

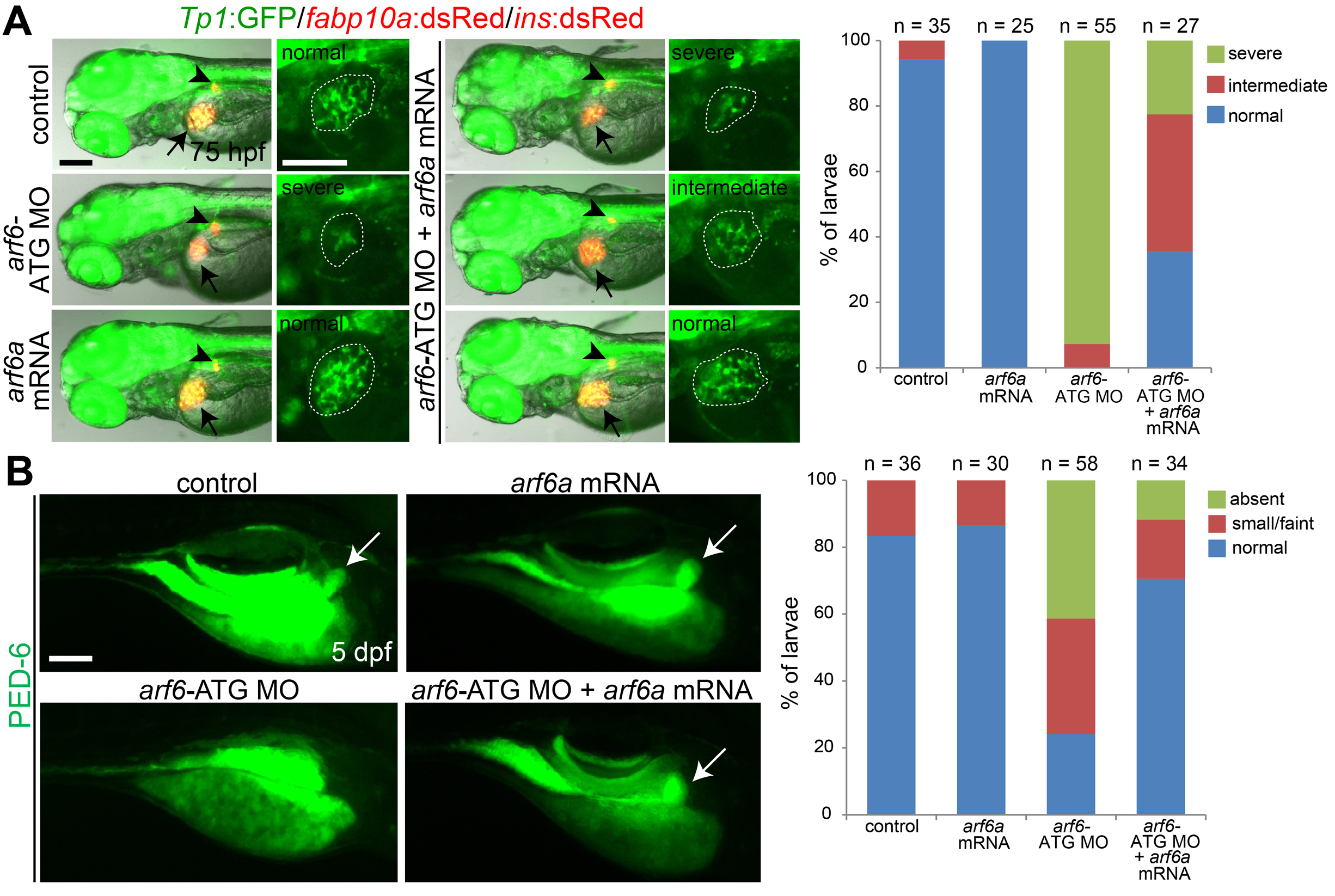

Supplement: S4 Fig — (A) The Tg(Tp1:GFP), Tg(fabp10a:dsRed), and Tg(ins:dsRed) lines were used to reveal the intrahepatic biliary structure, the liver, and the dorsal pancreas, respectively. Epifluorescence images showing the expression of these transgenes revealed that a defect in the intrahepatic biliary structure in arf6-ATG MO-injected larvae was partially rescued by arf6a mRNA injection. Based on the severity of the biliary defect, larvae were divided into three groups: normal, intermediate, and severe. Graph showing the percentage of larvae in each group. Arrows point to the liver; arrowheads point to the dorsal pancreas. Dotted lines outline the liver. Lateral views, anterior to the left. (B) Epifluorescence images showing PED-6 accumulation in the gallbladder revealed that the PED-6 accumulation defect in arf6-ATG MO-injected larvae was also partially rescued by arf6a mRNA injection. Based on PED-6 levels in the gallbladder, larvae were divided into three groups: absent, small/faint, and normal. Graph showing the percentage of larvae in each group. Arrows point to the gallbladder. Lateral views, anterior to the right. n indicates the number of larvae examined. Scale bars, 100 μm. (TIF) [file pone.0138381.s004.tif]

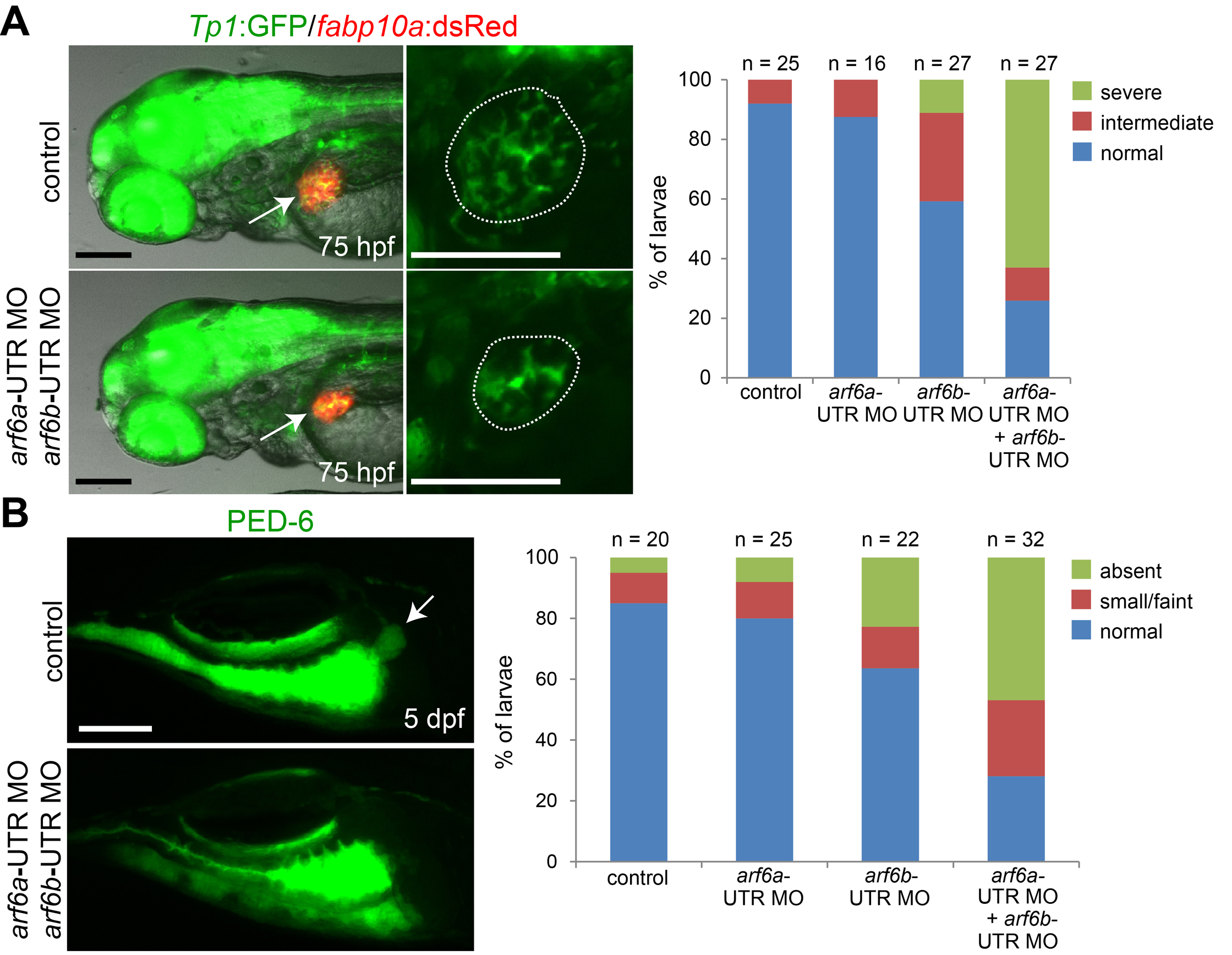

Supplement: S5 Fig — (A) The Tg(Tp1:GFP), Tg(fabp10a:dsRed), and Tg(ins:dsRed) lines were used to reveal the intrahepatic biliary structure, the liver, and the dorsal pancreas, respectively. Epifluorescence images showing the expression of these transgenes revealed that a severe biliary defect was observed more often in larvae co-injected with 3 ng of arf6a-UTR and 3 ng of arf6b-UTR MOs than in singly injected larvae. Based on the severity of the biliary defect, larvae were divided into three groups: normal, intermediate, and severe. Graph showing the percentage of larvae in each group. Arrows point to the liver; arrowheads point to the dorsal pancreas. Dotted lines outline the liver. Lateral views, anterior to the left. (B) Epifluorescence images showing PED-6 accumulation in the gallbladder. Based on PED-6 levels in the gallbladder, larvae were divided into three groups: absent, small/faint, and normal. Graph showing the percentage of larvae in each group. Arrows point to the gallbladder. Lateral views, anterior to the right. n indicates the number of larvae examined. Scale bars, 100 μm. (TIF) [file pone.0138381.s005.tif]

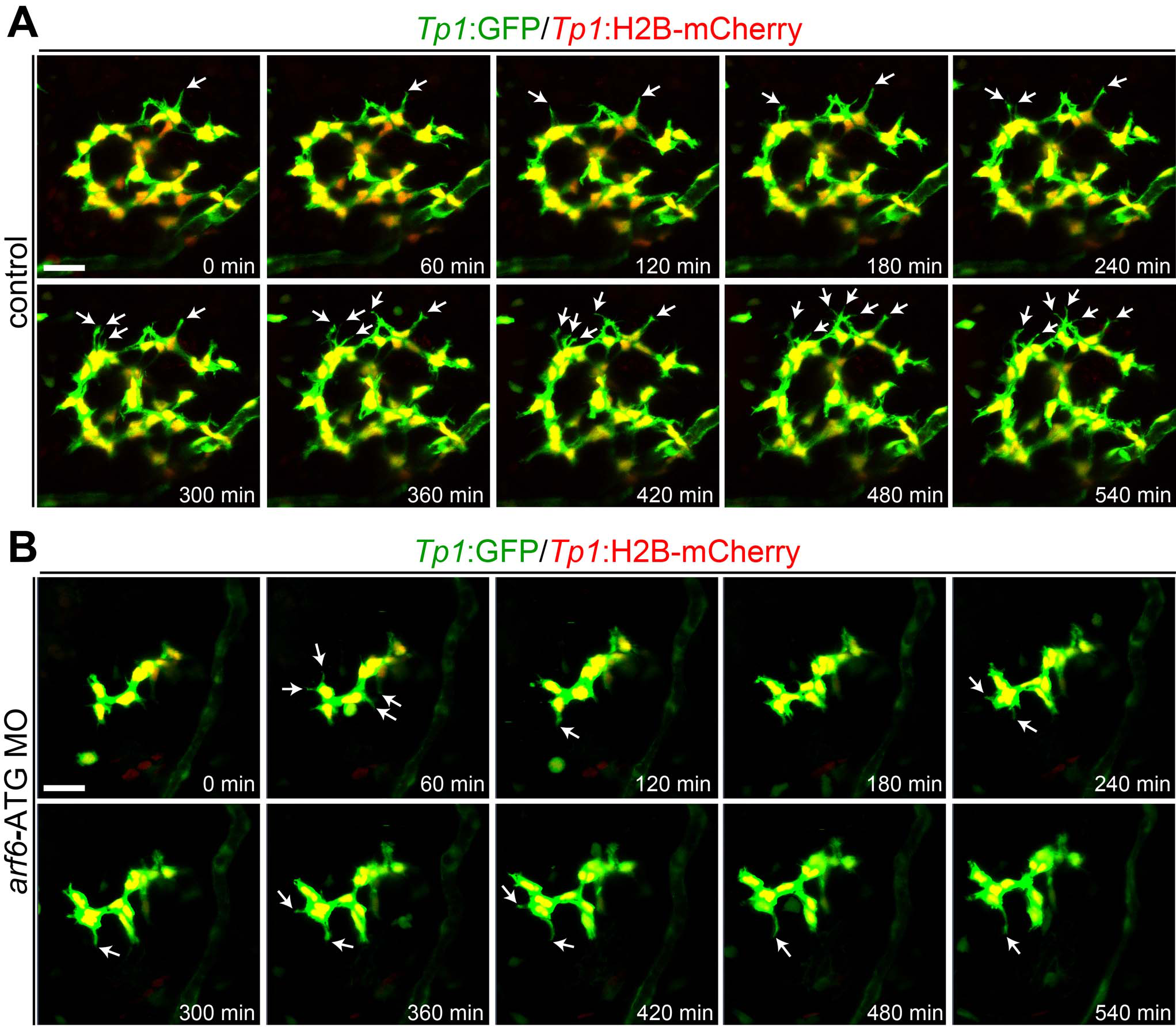

Supplement: S6 Fig — (A, B). Time-lapse confocal images showing BEC behaviors in arf6-ATG MO-injected and control larvae. The behaviors were assessed by Tp1:GFP (green) and Tp1:H2B-mCherry (red) expression in BEC cytoplasm and nuclei, respectively. Confocal images every 60 minutes from 74 to 84 hpf were presented. Arrows point to BEC filopodia. Scale bars, 25 μm. (TIF) [file pone.0138381.s006.tif]

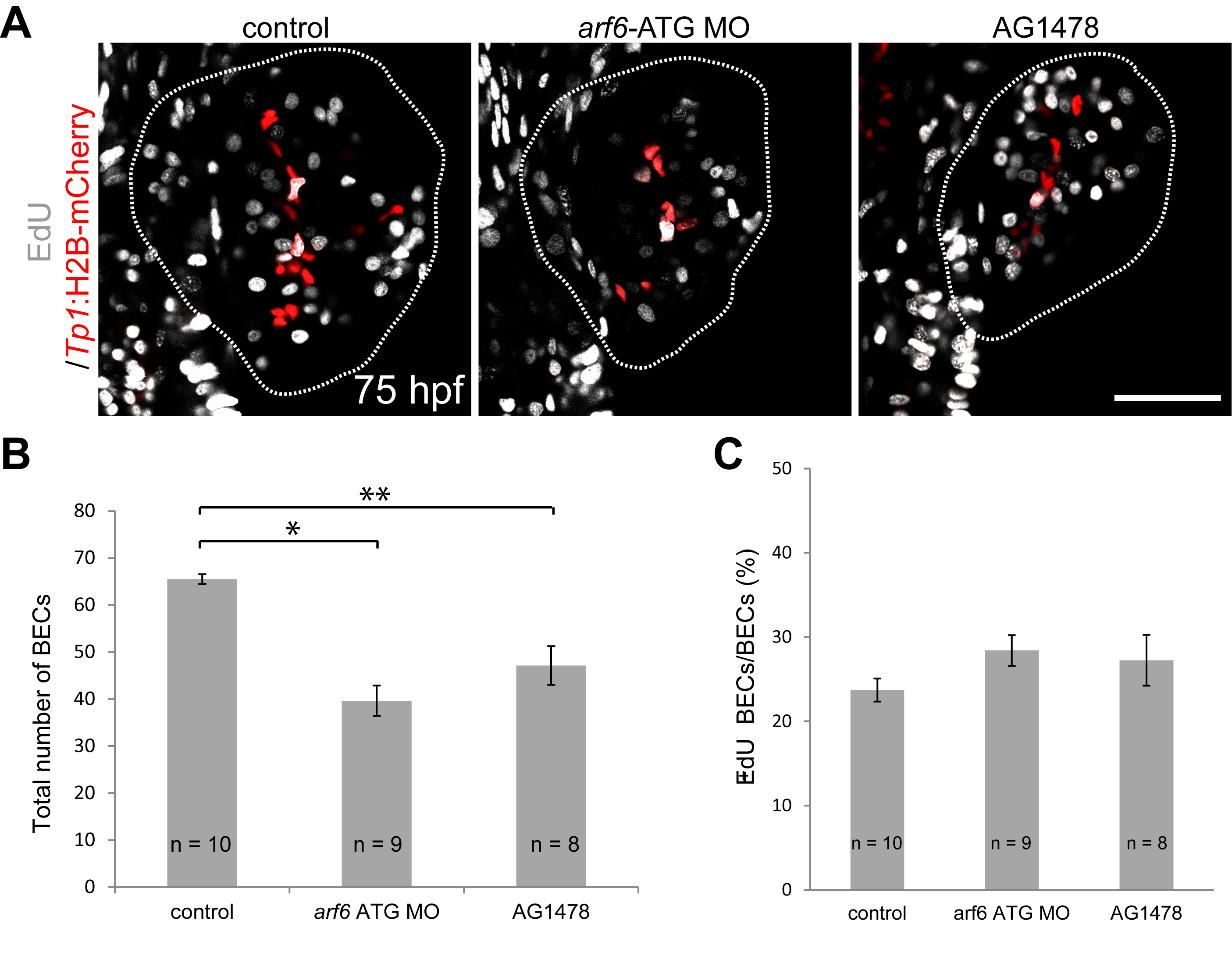

Supplement: S7 Fig — (A) Confocal images showing EdU+ proliferating cells (gray) and H2B-mCherry+ BECs (red) in the liver of control, arf6-ATG MO-injected, or AG1478-treated Tg(Tp1:H2B-mCherry) larvae. For EdU labeling, the larvae were treated with EdU for one hour prior to harvest. Dotted lines outline the liver. Scale bar, 50 μm. (B) Graph showing the total number of BECs in each liver. Asterisks indicate statistical significance: * p<0.0001, ** p<0.005. (C) Graph showing the percentage of EdU+ BECs among BECs. Error bars, ± SEM. n indicates the number of larvae examined. (TIF) [file pone.0138381.s007.tif]

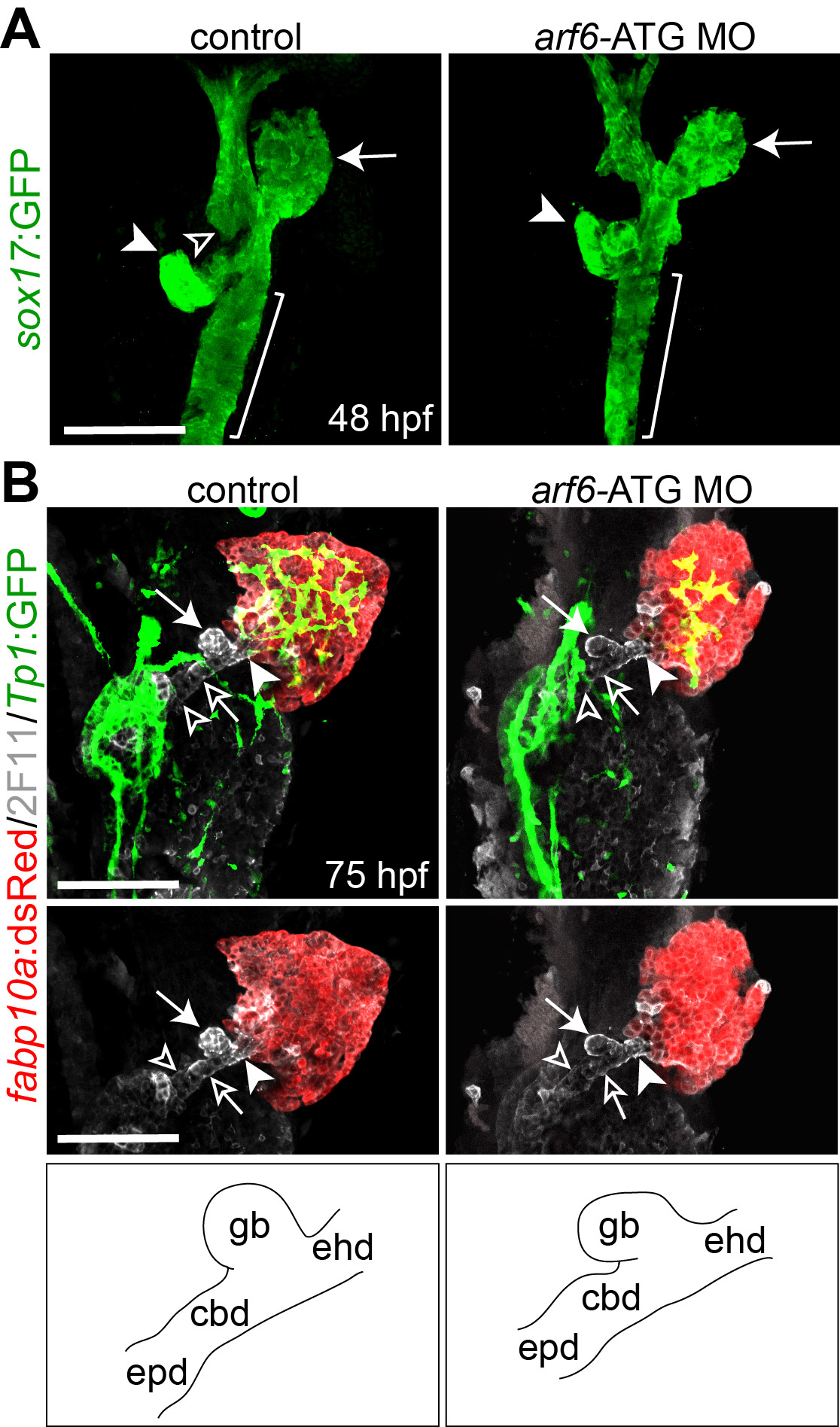

Supplement: S8 Fig — (A) Confocal images showing the endoderm and endoderm-derived organs at 48 hpf. The Tg(sox17:GFP) line was used to reveal the endoderm and endoderm-derived organs. Arrows, arrowheads, and open arrowhead point to the liver, the pancreas and the swim bladder, respectively; brackets mark the intestinal bulb. (B) Confocal images showing the hepatopancreatic ductal system at 75 hpf. Tg(Tp1:GFP);Tg(fabp10a:dsRed) larvae were processed for whole-mount immunostaining with 2F11 (gray), GFP (green), and dsRed (red) antibodies. In contrast to the intrahepatic biliary defect, the hepatopancreatic ductal system, revealed by 2F11 antibody, appeared to be normal in arf6-ATG MO-injected larvae. Arrows, arrowheads, open arrowheads, and open arrows point to the gallbladder (gb), the extrahepatic duct (ehd), the common bile duct (cbd), and the extrapancreatic duct (epd), respectively. The hepatopancreatic ductal system is schematically illustrated. Ventral views, anterior up. Scale bars, 50 μm. (TIF) [file pone.0138381.s008.tif]
