# Supplementary material for: The Role of ARF6 in Biliary Atresia
Source: PLoS One. 2015 Sep 17;10(9):e0138381. doi: 10.1371/journal.pone.0138381 (PMC4574480; doi:10.1371/journal.pone.0138381)
Supplement: S1 Table — (DOCX) [file pone.0138381.s009.docx]

| **Primer** | **Sequence** |
| --- | --- |
| *egfra* F | GCCTGATCTAAAGGACTGCAAAG |
| *egfra* R | GCCAGTAGACCTCCGACAA |
|  |  |
| *egfrb* F | CAAATGTGAAGGCTTGTGTCC |
| *egfrb* R | GATGTTGGTTGCGTTGACTG |
|  |  |
| *isecq1a* F | GCGGGAAATCTTCCTGTTTAATG |
| *isecq1a* R | GCATCCCGTACAGAGAGAAAG |
|  |  |
| *isecq1b* F | CCAGCATTCTCAGTCCATTCT |
| *isecq1b* R | GCATCTCCACCTGTTTGTCT |
|  |  |
| *arf6a* F | GAGCTGCACCGCATTATCA |
| *arf6a* R | CGTCTGGAAGGTCTTGTTTGT |
|  |  |
| *arf6b* F | CCCTCTGGCGACATTATTACAC |
| *arf6b* R | CCTCGTCTATGCGATCTCTATCT |
|  |  |
| *asap1a* F | ACAGACTCAGGATGAGGAGAA |
| *asap1a* R | CTGTAACCGCTCTGCTTACTC |
|  |  |
| *asap1b* F | GAGGATCTCACCAAAGCCATAA |
| *asap1b* R | CATGTCAGGATTCCCAGGTTAG |
|  |  |
| *rac1 F* | GGAATACATTCCCACTGTGTTTG |
| *rac1 R* | ATCCCACAATCCCAGGTTTAC |
|  |  |
| *gli1 F* | CCACTACAAGGCCAAGGTATT |
| *gli1 R* | GGTTTGGTTGTGTTCCCATTT |
|  |  |
| *gli2a F* | AAAAACAGGGCGGGACTACT |
| *gli2a R* | ATGCTGGGTTGGAGGTACAG |
|  |  |
| *ptch1 F* | GGTTACCATGGATGGCTTTG |
| *ptch1 R* | TCAGCATCAAAAGTGGCTTG |
|  |  |
| *eef1a1l1 F* | GGCAGACCGTTGCTGTCGGCG |
| *eef1a1l1 R* | GGTTGGGAAGAACACGCCGCAACCT |
