# Supplementary material for: The Role of ARF6 in Biliary Atresia
Source: PLoS One. 2015 Sep 17;10(9):e0138381. doi: 10.1371/journal.pone.0138381 (PMC4574480; doi:10.1371/journal.pone.0138381)
Supplement: S4 Table — (DOCX) [file pone.0138381.s012.docx]

| **Gene** | **Batch 1** | **Batch 2** | **Batch 3** |
| --- | --- | --- | --- |
| *ptch1* | 2.13 | 1.94 | 1.49 |
| *gli1* | -1.52 | 1.82 | 1.82 |
| *gli2a* | 3.71 | -1.19 | 2 |
